# Unveiling the mechanism of broad‐spectrum blast resistance in rice: The collaborative role of transcription factor OsGRAS30 and histone deacetylase OsHDAC1

**DOI:** 10.1111/pbi.14299

**Published:** 2024-01-31

**Authors:** Jiaqi Hou, Huangzhuo Xiao, Peng Yao, Xiaoci Ma, Qipeng Shi, Jin Yang, Haoli Hou, Lijia Li

**Affiliations:** ^1^ State Key Laboratory of Hybrid Rice, College of Life Sciences Wuhan University Wuhan China

**Keywords:** OsHDAC1, OsGRAS30, ChIP‐Seq, transcriptome, H3K27ac, rice broad‐spectrum blast resistance

## Abstract

Rice blast, caused by *Magnaporthe oryzae*, significantly impacts grain yield, necessitating the identification of broad‐spectrum resistance genes and their functional mechanisms for disease‐resistant crop breeding. Here, we report that rice with knockdown *OsHDAC1* gene expression displays enhanced broad‐spectrum blast resistance without effects on plant height and tiller numbers compared to wild‐type rice, while rice overexpressing *OsHDAC1* is more susceptible to *M. oryzae*. We identify a novel blast resistance transcription factor, OsGRAS30, which genetically acts upstream of OsHDAC1 and interacts with OsHDAC1 to suppress its enzymatic activity. This inhibition increases the histone H3K27ac level, thereby boosting broad‐spectrum blast resistance. Integrating genome‐wide mapping of OsHDAC1 and H3K27ac targets with RNA sequencing analysis unveils how OsHDAC1 mediates the expression of *OsSSI2*, *OsF3H*, *OsRLR1* and *OsRGA5* to regulate blast resistance. Our findings reveal that the OsGRAS30–OsHDAC1 module is critical to rice blast control. Therefore, targeting either OsHDAC1 or OsGRAS30 offers a promising approach for enhancing crop blast resistance.

## Introduction

Rice (*Oryza sativa* L.) is one of the world's most important cereal crops, feeding half of the global population (Zhang *et al*., 2020). Rice blast is caused by the pathogenic fungus *Magnaporthe oryzae* and is one of the most serious diseases affecting rice, often causing serious decreases in rice production (Chakraborty *et al*., 2021). Plants have developed two layers of defence against pathogens, including pathogen‐associated molecular pattern‐triggered immunity (PTI) and effector‐triggered immunity (ETI) (Jones and Dangl, 2006). PTI occurs at the plasma membrane surface and is controlled by pattern recognition receptors (Couto and Zipfel, 2016). Pathogens secrete effector molecules encoded by virulence genes into plant cells to induce pathogen virulence and attenuate plant PTI (Xin *et al*., 2018). Such pathogen effectors are detected by intracellular plant receptors known as nucleotide‐binding leucine‐rich repeat resistance (R) proteins, activating ETI (Thordal‐Christensen, 2020). To date, over 100 blast R genes in the rice genome and 24 virulence genes in *M. oryzae* have been identified (Li *et al*., 2019). Blast R genes interact directly or indirectly with pathogenic effectors following *M. oryzae* infection, to trigger changes in the expression of defence response‐related genes, triggering diverse physiological events including hormone signalling activation, hypersensitivity reactions, biosynthesis of chemical molecules that degrade pathogen cells and production of reactive oxygen species (Liu *et al*., 2021). However, such resistance is race‐specific and is easily lost due to the rapid evolution of pathogen virulence genes (Deng and Naqvi, 2019). In contrast, non‐race‐specific resistance is broad‐spectrum and may fight crop diseases more effectively. To date, a few genes conferring broad‐spectrum resistance to pathogens have been identified in plants, including *RPW8.1* and *RPW8.2* for broad‐spectrum resistance to powdery mildew in *Arabidopsis* (Xiao *et al*., 2001; Yang *et al*., 2023; Zhao *et al*., 2021, 2023), *STV11* for durable resistance to rice stripe virus (Wang *et al*., 2014), *bsr‐d1*, *OsUBC45* and *RBL1* for broad‐spectrum blast resistance in rice (Li *et al*., 2017; Sha *et al*., 2023; Wang *et al*., 2023), *Lr34* and *TapsIPK1* for broad‐spectrum resistance to rust fungi in wheat (Fukuoka *et al*., 2009; Wang *et al*., 2022) and *ZmGLK36* for broad‐spectrum resistance to maize rough dwarf disease (Xu *et al*., 2023). In plants, a growth–defence tradeoff exists due to resource constraints, which requires plants to prioritize either growth or defence according to changes in external and internal factors (Huot *et al*., 2014). This tradeoff means that most defence genes also affect agricultural traits (He *et al*., 2022). Thus, broad‐spectrum genes with no or little effect on crop growth should be identified and integrated into breeding strategies to optimize the growth–defence balance, maximize crop yields and meet rising global food demands.

Recent reports have shown that epigenetic modification‐mediated transcription regulation is involved in many biological processes in plants (Chang *et al*., 2020; Chen *et al*., 2020; Liang *et al*., 2020; Yang *et al*., 2022). Histone acetylation is one of the most extensively characterized epigenetic modifications and is generally associated with active transcription (Shvedunova and Akhtar, 2022). Histone deacetylases (HDACs) regulate histone acetylation levels and gene transcription by removing acetyl moieties from the acetylated lysines of histones, which are added by histone acetyltransferases (Kumar *et al*., 2021), crucial players in plant growth and development as well as responses to environmental stimuli (Chen *et al*., 2020). In addition, HDACs are involved in disease resistance in some plant species. For example, *Arabidopsis* HDA6 suppresses the expression of defence‐related genes (Wang *et al*., 2017; Wu *et al*., 2021). HDA19 is required for salicylic acid (SA)‐mediated defence responses (Choi *et al*., 2012), and HDA19 can interact with WRKY38 or WRKY62 to regulate plant basal defence responses (Kim *et al*., 2008). HD2B is involved in the reprogramming of defence gene expression and innate immunity (Latrasse *et al*., 2017). In rice, HDA701 negatively regulates broad‐spectrum resistance to rice pathogens by modulating histone H3K9 acetylation of defence‐related genes (Chen *et al*., 2022a). SRT702 negatively regulates resistance to rice blast, false smut and bacterial blight by modulating histone H4K5 and H4K8 acetylation to affect the expression of disease‐resistance genes (Chen *et al*., 2024). HDT701 negatively regulates plant innate immunity by modulating histone H4 acetylation of defence‐related genes (Ding *et al*., 2012), and the RNase P protein subunit Rpp30 can target HDT701, conferring resistance to fungal and bacterial pathogens (Li *et al*., 2021). In wheat, HOS15 can interact with HDA6 to regulate defence responses to powdery mildew (Zhi *et al*., 2020). However, the molecular mechanisms of HDAC activity in plant immunity remain largely unknown.

Seventeen HDAC genes have been identified in the rice genome (Hou *et al*., 2021). Here, we demonstrate OsHDAC1 as a negative regulator of immunity against rice blast with no effects on plant height and tiller numbers and identify a novel GRAS transcription factor OsGRAS30, which can interact with OsHDAC1 and suppress its activity, thereby enhancing rice broad‐spectrum blast resistance. Further, we have found that OsHDAC1 suppresses the transcription of blast resistance‐related genes by binding their promoters and reducing histone H3K27 acetylation. Moreover, we show that chemical HDAC inhibitors confer rice blast resistance, providing a novel strategy for controlling rice blast. Overall, our data reveal the functional characteristics of the OsGRAS30–OsHDAC1 module in mediating broad‐spectrum blast resistance in rice.

## Results

### OsHDAC1 negatively regulates broad‐spectrum resistance to *M. Oryzae* in rice

**Figure 1:**
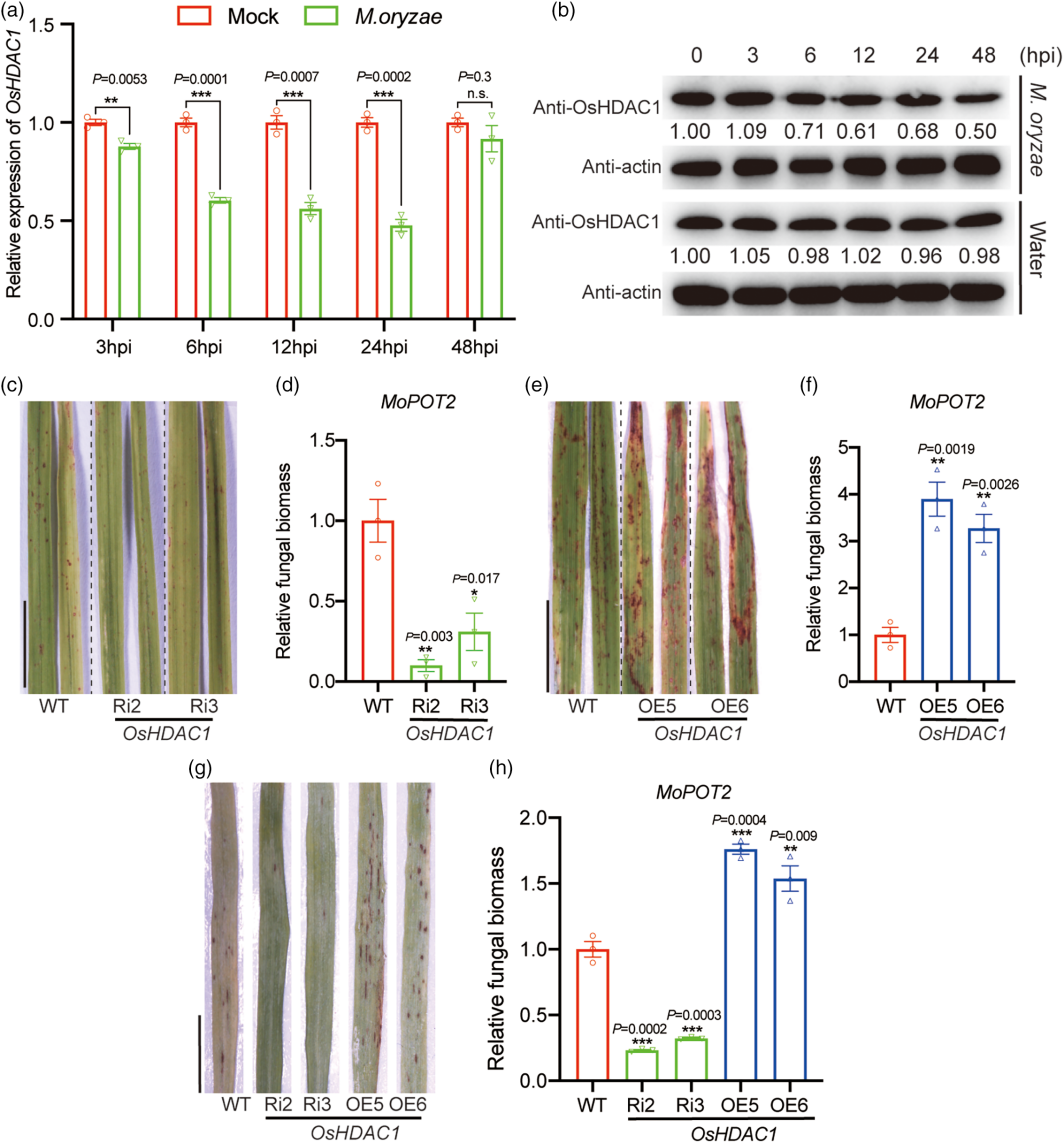
OsHDAC1 regulates blast resistance in rice. (a) Transcript variation of *OsHDAC1* in response to *M. oryzae* Guy11 treatment was analysed by RT–qPCR using total RNA extracted from leaves at 3, 6, 12, 24 or 48‐h post‐infection (hpi). Gene expression levels in the WT without *M. oryzae* treatment were set to 1.00. Values are means ± SEM (*n* = 3). Experiments were repeated three times with similar results. (b) Protein level of OsHDAC1was reduced in response to *M. oryzae* Guy11 treatment, as shown by immunoblotting analysis. Water treatment was used as a control, and the actin protein was applied as an equal loading control. (c) Disease symptoms after spray‐inoculation with a spore suspension of *M. oryzae* Guy11 for 5 days post inoculation (dpi) in *OsHDAC1* RNAi lines and WT. Scale bar = 1 cm. Representative data from three independent experiments. (d) Relative fungal biomass in *OsHDAC1* RNAi lines compared with WT determined by qPCR for the *M. oryzae Pot2* (*MoPot2*) gene normalized to rice *Ubiquitin*. Fungal biomass in the WT was set to 1.00. Values are means ± SEM (*n* = 3). Experiments were repeated three times with similar results. (e) Disease symptoms after spray‐inoculation with a spore suspension of *M. oryzae* Guy11 for 5 dpi in *OsHDAC1* OE lines and WT. Scale bar = 1 cm. Representative data from three independent experiments. (f) Relative fungal biomass in *OsHDAC1* OE lines compared with WT determined using qPCR for the *MoPot2* gene normalized to rice *Ubiquitin*. Fungal biomass in the WT was set to 1.00. Values are means ± SEM (*n* = 3). Experiments were repeated three times with similar results. (g) Disease symptoms of the WT, *OsHDAC1* OE and RNAi rice leaves inoculated with mixed isolates including AH4, Sc09‐153‐07, TM3‐2, RB1, RB3, NC1, HLJ1‐3, HLJ09‐17‐1 and HLJ5‐3. Scale bar = 1 cm. Representative data from three independent experiments. (h) Relative fungal biomass in *OsHDAC1* RNAi and OE lines compared with WT determined using qPCR for the *MoPot2* gene normalized to rice *Ubiquitin*. Fungal biomass in the WT was set to 1.00. Values are means ± SEM (*n* = 3). Experiments were repeated three times with similar results. Asterisks represent significant differences determined by Student's *t‐test* (*P < 0.05; **P < 0.01; ***P < 0.001). WT, wild‐type; Ri, RNA interference; OE, overexpression.

Recently, chromatin histone modifications have been reported to play a role in the regulation of plant disease defences (Chen *et al*., 2022a; Li *et al*., 2021; Niu *et al*., 2019). In our previous work, to examine the function of OsHDAC1, we constructed *OsHDAC1* RNA interference (RNAi) lines in rice, as homozygous *OsHDAC1* mutant seeds could not be obtained, and also generated overexpression (OE) transgenic plants (Chung *et al*., 2009; Hou *et al*., 2022). In this study, to explore whether OsHDAC1 is involved in rice blast resistance, we first challenged rice plants with the virulent *M. oryzae* Guy11. To detect changes in OsHDAC1 protein levels in these rice plants, we produced an OsHDAC1‐specific antibody (Figure S1). Analysis of changes in *OsHDAC1* expression after *M. oryzae* infection showed that the transcript (Figure 1a) and protein (Figure 1b) levels of *OsHDAC1* were downregulated. To investigate the possible involvement of OsHDAC1 in blast resistance, two *OsHDAC1* RNAi lines and two *OsHDAC1* OE lines, along with wild‐type (WT) Nipponbare rice, were inoculated with *M. oryzae* Guy11 by spraying with conidia. The two *OsHDAC1* RNAi lines (Ri2 and Ri3) developed only small, scattered lesions, while WT Nipponbare showed typical blast lesions (Figure 1c), indicating that silencing of OsHDAC1 enhances resistance to blast. Fungal DNA quantification showed that the two *OsHDAC1* RNAi lines harboured only 10% and 30% as much *M. oryzae* as WT, respectively (Figure 1d). In contrast, the *OsHDAC1* OE5 and OE6 lines had larger lesions compared with WT (Figure 1e). The two OE lines had significantly greater fungal DNA content, which was increased by approximately 3‐ to 4‐fold (Figure 1f). We then inoculated the *OsHDAC1* RNAi and OE lines and WT by spraying conidia from mixed *M. oryzae* isolates, including AH4, Sc09‐153‐07, TM3‐2, RB1, RB3, NC1, HLJ1‐3, HLJ09‐17‐1 and HLJ5‐3 from different regions of China (Figure S2). The *OsHDAC1* RNAi lines showed enhanced resistance to multiple virulent isolates of *M. oryzae*, whereas the OE lines showed higher susceptibility, compared with WT (Figure 1g). Relative fungal biomass was reduced by approximately 3‐fold in the RNAi lines and increased by 1.7‐ and 1.5‐fold in the OE lines (Figure 1h). In addition, field tests demonstrated that the *OsHDAC1* OE and RNAi lines had no effects on plant height, tiller numbers, 1000 seed weight and seed setting rate (Figure S3). These results indicate that OsHDAC1 negatively regulates rice broad‐spectrum blast resistance.

### OsGRAS30 interacts with OsHDAC1

**Figure 2:**
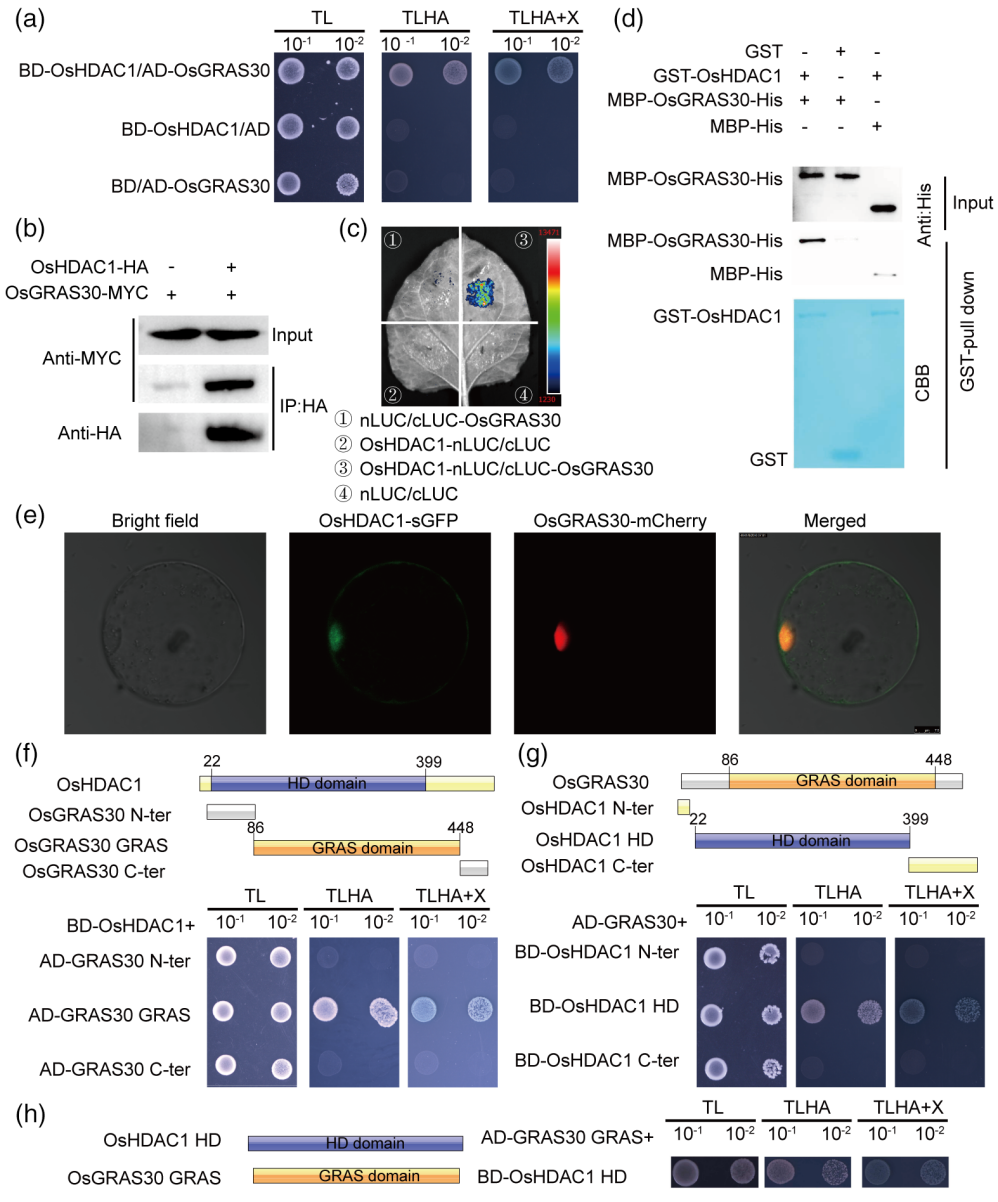
OsHDAC1 interacts with OsGRAS30. (a) Yeast two‐hybrid (Y2H) assay showing OsHDAC1 and OsGRAS30 interaction in yeast. Empty BD and AD were used as the negative controls. (b) Coimmunoprecipitation assay showed that OsHDAC1 interacted with OsGRAS30 in rice protoplast. + or ‐, presence or absence of protein. (c) Split‐luciferase complementation assay showing the interaction between OsHDAC1 and OsGRAS30 in *N. benthamiana* leaves. Empty nLUC and cLUC were used as negative controls. (d) GST pull‐down assay showing the interaction between OsHDAC1 and OsGRAS30 *in vitro*. GST‐OsHDAC1 and GST were stained with CBB as loading controls. GST and MBP tags were used as the negative control. + or –, presence or absence of protein. (e) Confocal microscopy result of OsHDAC1‐sGFP co‐localizing with OsGRAS30‐mCherry in rice protoplasts. Scale bar = 7.5 μm. (f–h) Y2H assays showing the interaction between different regions of OsHDAC1 and different regions of OsGRAS30 in yeast. Schematic of the domain structure of OsHDAC1 (f) and OsGRAS30 (g) are indicated. SD, synthetic dropout medium; BD, binding domain; AD, activation domain; TL, SD medium lacking leucine and tryptophan; TLHA, SD medium lacking leucine, tryptophan, histidine and adenine; X, X‐α‐gal. HA, HA epitope tag sequence; MYC, MYC epitope tag sequence; GST, GST epitope tag sequence; His, His epitope tag sequence; MBP, MBP epitope tag sequence; CBB, Coomassie brilliant blue; LUC, luciferase gene; N‐ter, N‐terminal region; C‐ter, C‐terminal region; HD, histone deacetylase domain; GRAS, GRAS domain.

To gain mechanistic insights into OsHDAC1 function, we attempted to identify proteins interacting with OsHDAC1 in a yeast screening experiment. We identified the gene *LOC_Os05g49930*, named *OsGRAS30*, which belongs to the GRAS transcription factor family and interacts with OsHDAC1 (Figure 2a). Previous studies indicated that overexpression of the GRAS transcription factor family proteins GIGR2 and OsSLR1 promotes resistance to *M. oryzae* (De‐Vleesschauwer *et al*., 2016; Tanabe *et al*., 2016). Therefore, we speculated that OsGRAS30 might be involved in OsHDAC1‐mediated *M. oryzae* resistance. To confirm the OsHDAC1–OsGRAS30 interaction *in vivo*, OsHDAC1‐HA and OsGRAS30‐Myc were co‐expressed in rice protoplasts and subjected to coimmunoprecipitation (Co‐IP) analysis. OsGRAS30‐Myc could be co‐purified with OsHDAC1‐HA using an anti‐HA antibody, but no OsGRAS30‐Myc was obtained from the control (Figure 2b). We conclude that OsHDAC1 interacts with OsGRAS30 *in vivo*. Further, in a split‐luciferase (Split‐LUC) complementation imaging assay, we transiently co‐infiltrated *Nicotiana benthamiana* leaves with OsHDAC1‐nLUC and cLUC‐OsGRAS30 fusion constructs, and LUC fluorescence was apparent in *N. benthamiana* leaves, demonstrating OsHDAC1–OsGRAS30 interaction *in planta* (Figure 2c). To confirm the OsHDAC1–OsGRAS30 interaction *in vitro*, we expressed and purified recombinant GST‐OsHDAC1 and MBP‐OsGRAS30‐His protein from *Escherichia coli* and performed an *in vitro* pull‐down assay. MBP‐OsGRAS30‐His was pulled down by GST‐OsHDAC1 but not by GST itself (Figure 2d), suggesting that MBP‐OsGRAS30‐His interacts with GST‐OsHDAC1 *in vitro*. In addition, laser confocal microscopy showed that OsHDAC1‐sGFP and OsGRAS30‐mCherry were co‐localized in the nucleus of rice protoplasts (Figure 2e).

We attempted to delineate the interaction interface by testing three OsHDAC1 fragments for interaction with three OsGRAS30 fragments in a yeast two‐hybrid (Y2H) assay. First, we divided OsGRAS30 into three parts: the N‐terminal region (amino acids 1–85), GRAS domain (amino acids 86–448) and C‐terminal region (amino acids 449–501). The results showed that the GRAS domain region of OsGRAS30 was important for its interaction with OsHDAC1 (Figure 2f). Then, we divided OsHDAC1 into three parts: the N‐terminal region (amino acids 1–22), histone deacetylase domain (amino acids 22–399) and C‐terminal region (amino acids 400–518). As shown in Figure 2g, the histone deacetylase domain of OsHDAC1 interacted with OsGRAS30. We further found that the histone deacetylase domain of OsHDAC1 interacted with the GRAS domain region of OsGRAS30 (Figure 2h). These results indicate that the histone deacetylase domain of OsHDAC1 and the GRAS domain region of OsGRAS30 are essential for their interaction.

### OsGRAS30 functions as a positive regulator of rice blast resistance

**Figure 3:**
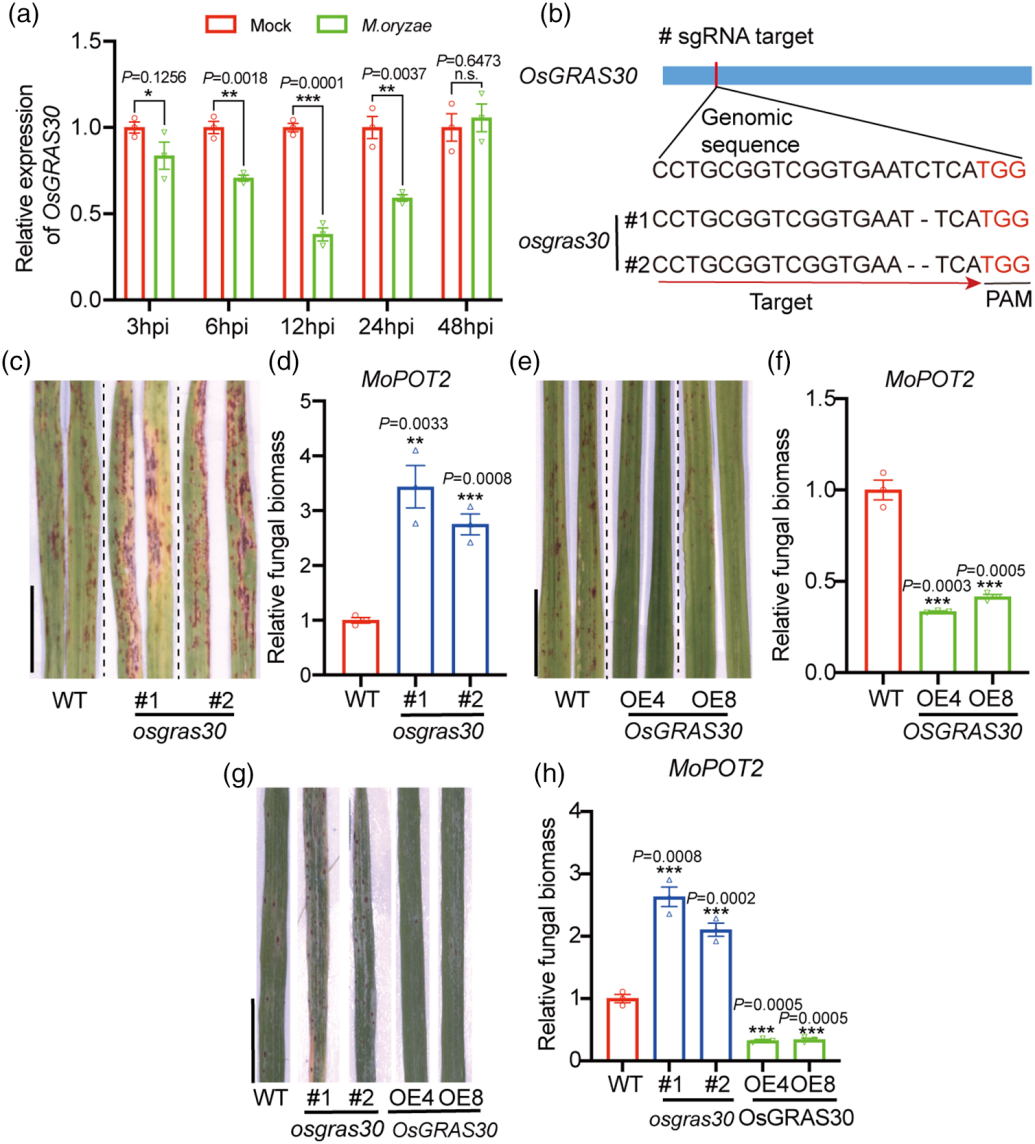
OsGRAS30 is correlated with blast resistance in rice. (a) Transcript variation of *OsGRAS30* in response to *M. oryzae*. Total RNA was extracted from leaves after 3, 6, 12, 24 or 48‐h *M. oryzae* infection and subjected to RT–qPCR. Expression levels of genes in the WT without *M. oryzae* treatment were set to 1.00. Values are mean ± SEM (*n* = 3). Experiments were repeated three times with similar results. (b) Gene structure and mutation sites of *OsGRAS30*. sgRNA, small guide RNA; PAM, protospacer adjacent motif. (c) Disease symptoms after spray‐inoculation with a spore suspension of *M. oryzae* for 5 dpi in *OsGRAS30* mutants and WT. Scale bar = 1 cm. Representative data from three independent experiments. (d) Relative fungal biomass in *OsGRAS30* mutants compared with WT determined using qPCR for the *MoPot2* normalized to rice *Ubiquitin*. Fungal biomass in the WT treated with *M. oryzae* was set to 1.00. Values are means ± SEM (*n* = 3). Experiments were repeated three times with similar results. (e) Disease symptoms after spray‐inoculation with a spore suspension of *M. oryzae* for 5 dpi in *OsGRAS30* OE lines and WT. Scale bar = 1 cm. Representative data from three independent experiments. (f) Relative fungal biomass in *OsGRAS30* OE lines compared with WT determined by qPCR for the *MoPot2* normalized to rice *Ubiquitin*. Fungal biomass in the WT treated with *M. oryzae* was set to 1.00. Values are means ± SEM (*n* = 3). Experiments were repeated three times with similar results. (g) Disease symptoms of the *OsGRAS30* mutant and OE lines in rice leaves inoculated with mixed isolates including AH4, Sc09‐153‐07, TM3‐2, RB1, RB3, NC1, HLJ1‐3, HLJ09‐17‐1 and HLJ5‐3 compared with WT. Scale bar = 1 cm. Representative data from three independent experiments. (h) Relative fungal biomass determined in *OsGRAS30* mutant and OE lines compared with WT determined using qPCR for the *MoPot2* gene normalized to rice *Ubiquitin*. Values are means ± SEM (*n* = 3). Experiments were repeated three times with similar results. Asterisks mark significant changes compared with WT without *M. oryzae* treatment (a) or WT with *M. oryzae* treatment (d, f and h) based on Student's *t‐test*: *P < 0.05, **P < 0.01, ***P < 0.001. WT, wild‐type; OE, overexpression.

To investigate the role of OsGRAS30 in the response to *M. oryzae*, we first assessed the change in its expression levels after blast infection. The RNA level began to decrease at 3 h post‐infection (hpi) with blast and was further reduced at 12 hpi, reaching 40% of the level in plants with no blast infection (Figure 3a). We then generated loss‐of‐function and gain‐of‐function transgenic plants. We used clustered regularly interspaced short palindromic repeats (CRISPR)/Cas9 technology to create *OsGRAS30*‐knockout mutants, with one site in the exon selected for guide RNA (gRNA) design, as shown in Figure 3b. DNA sequencing revealed that among the mutant lines isolated in this study, *osgras30* #1 has a single nucleotide deletion, and *osgras30 #*2 has a two‐nucleotide deletion at the gRNA site (Figure 3b). Each mutation causes a frameshift in the OsGRAS30‐coding sequence. We generated rice transgenic rice plants overexpressing *OsGRAS30*‐MYC using the pCXUN vector containing *OsGRAS30* cDNA fused to the MYC tag driven by the *Ubiquitin* promoter in the WT background (Figure S4a). Of 10 OE lines, 4 exhibited increased *OsGRAS30* RNA levels to varying degrees (Figure S4b). We selected lines OE4 and OE8, which displayed the highest *OsGRAS30* RNA levels, for further study.

Next, two *OsGRAS30* mutant lines (*osgras30* #1 and #2), the *OsGRAS30* OE lines, and WT were inoculated with blast by spraying Guy11 conidia. The results showed that the *osgras30* #1 and *osgras30* #2 lines exhibited higher susceptibility to blast treatment compared with WT plants (Figure 3c), and the fungal DNA contents in the *OsGRAS30* mutant lines were increased by 2.7‐ and 3.3‐fold compared with WT (Figure 3d). In contrast, we observed enhanced resistance to blast after inoculating the *OsGRAS30* OE4 and OE8 lines and WT (Figure 3e). These two OE lines contained lower fungal DNA content than that of WT (Figure 3f). We further inoculated the *OsGRAS30* mutant and OE lines along with WT by spraying conidia from mixed *M. oryzae* isolates including AH4, Sc09‐153‐07, TM3‐2, RB1, RB3, NC1, HLJ1‐3, HLJ09‐17‐1 and HLJ5‐3 (Figure S2). The *OsGRAS30* OE lines showed enhanced resistance to multiple virulent isolates of *M. oryzae*, whereas the mutant lines showed higher susceptibility, compared with WT (Figure 3g). Relative fungal biomass increased by 2‐ and 2.5‐fold in the mutant lines and decreased by 3‐fold in the OE lines (Figure 3h). Taken together, these data from the mutant and OE lines suggest that the *OsGRAS30* gene positively regulates broad‐spectrum resistance to rice blast.

### *OsHDAC1* acts genetically downstream of *OsGRAS30*

**Figure 4:**
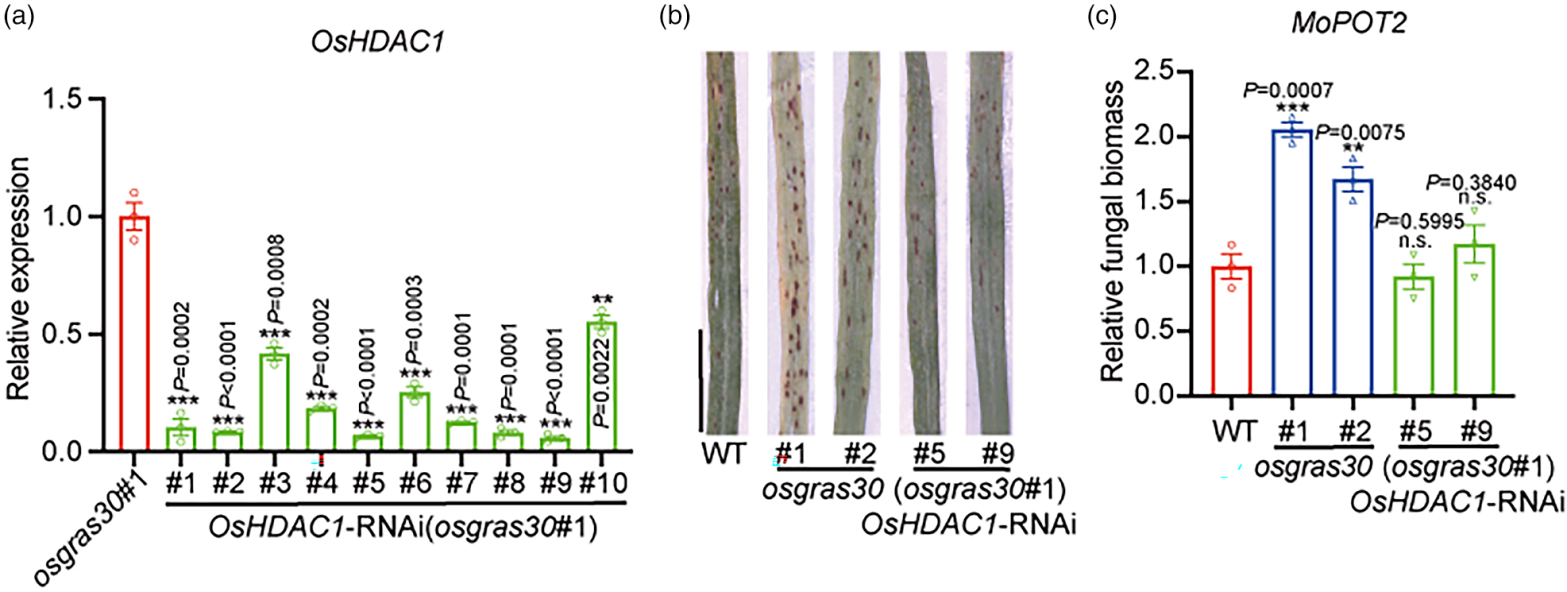
OsGRAS30 promotes blast resistance in an OsHDAC1‐dependent manner. (a) Expression levels of *OsHDAC1* in *OsHDAC1* RNAi lines (from #1 to #10) with the *osgras30* #1 background. Total RNA was extracted from leaves and subjected to RT–qPCR. Expression levels of *OsHDAC1* in the *osgras30* #1 plants were set to 1.00. Values are mean ± SEM (*n* = 3). Experiments were repeated three times with similar results. (b) Disease symptoms after spray‐inoculation with a spore suspension of *M. oryzae* for 5 dpi in *OsHDAC1* RNAi lines with the *osgras30* #1 background compared with WT and *OsGRAS30* mutants. Scale bar = 1 cm. Representative data from three independent experiments. (c) Relative fungal biomass in *OsHDAC1* RNAi lines with the *osgras30* #1 background compared with WT and *OsGRAS30* mutants determined using qPCR for the *MoPot2* gene normalized to rice *Ubiquitin*. Fungal biomass in the WT plants infected with *M. oryzae* was set to 1.00. Values are means ± SEM (*n* = 3). Experiments were repeated three times with similar results. Asterisks mark significant changes compared with *osgras30* #1(a and c) based on Student's *t‐test*: **P < 0.01, ***P < 0.001.

To analyse the genetic interactions between *OsGRAS30* and *OsHDAC1*, we used *Agrobacterium tumefaciens* strain EHA105 containing *OsHDAC1*‐RNAi constructs (Hou *et al*., 2022) to transform the background of *osgras30 #*1 plants and thereby generated homozygous double *OsHDAC1* RNAi/*osgras30* lines. Through this process, we obtained 10 putative *OsHDAC1* RNAi lines of *osgras30 #*1 rice with reduced *OsHDAC1* expression; *OsHDAC1* RNA levels were reduced by approximately 90% in the *OsHDAC1* RNAi #5 and #9 lines compared with the *osgras30 #*1 line (Figure 4a). Then, two *OsHDAC1* RNAi lines with the *osgras30 #*1 background, the *osgras30* mutants and WT were inoculated with conidia from mixed *M. oryzae* isolates. The results indicated that the *OsHDAC1* RNAi #5 and #9 lines in the *osgras30 #*1 background showed enhanced resistance to multiple virulent isolates of *M. oryzae* compared with *OsGRAS30* mutants, whereas *OsHDAC1* RNAi #5 and #9 lines in the *osgras30 #*1 background showed similar blast resistance phenotype observed in the WT (Figure 4b). Measurement of *M. oryzae* DNA content confirmed the phenotype results (Figure 4c). This demonstrates that *OsGRAS30* acts, at least in part, genetically upstream of *OsHDAC1* to positively regulate blast resistance.

### OsGRAS30 represses OsHDAC1 enzyme activity to induce H3K27ac hypoacetylation

**Figure 5:**
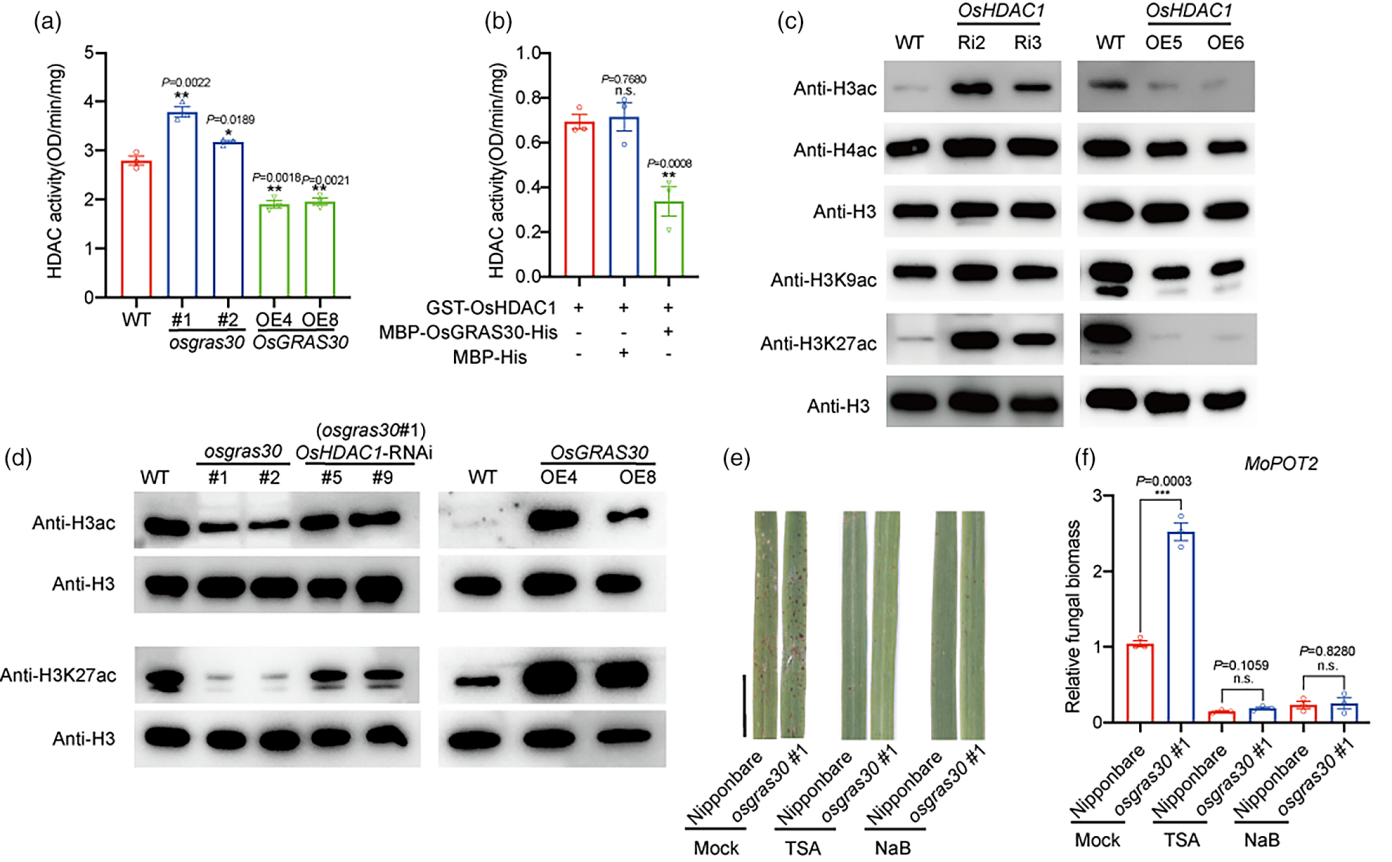
OsGRAS30 represses OsHDAC1 to induce H3K27ac hypoacetylation. (a) *In vivo* HDAC activity assays in WT, *OsGRAS30* mutant and OE lines. Values are mean ± SEM (*n* = 3). Experiments were repeated three times with similar results. (b) *In vitro* analysis of the effect of OsGRAS30 on OsHDAC1 activity. Values are mean ± SEM (*n* = 3). + or –, presence or absence of proteins. Experiments were repeated three times with similar results. (c) Overall H3ac, H4ac, H3K9ac and H3K27ac levels in WT, *OsHDAC1* RNAi and OE lines using immunoblotting. Histone H3 was applied as an equal loading control. Representative data from three independent experiments. (d) Immunoblotting analysis of the overall H3ac, H4ac, H3K9ac and H3K27ac levels in WT, *OsGRAS30* mutant lines, and *OsHDAC1* RNAi lines in the background of *osgras30* #1 and *OsGRAS30* OE lines (line numbers are given above the graph). H3 protein was applied as an equal loading control. Representative data from three independent experiments. (e) Disease symptoms after spray‐inoculation with a spore suspension of *M. oryzae* for 5 dpi in *osgras30#1* and WT treated with HDAC inhibitors including TSA and NaB. Untreated was used as a control. Scale bar = 1 cm. Representative data from three independent experiments. (f) Relative fungal biomass was determined using qPCR for the *MoPot2* gene normalized to rice *Ubiquitin*. Values are means ± SEM (*n* = 3). Experiments were repeated three times with similar results. Asterisks mark significant changes compared with WT (a and b) or WT with *M. oryzae* treatment (f) based on Student's *t* test: *P < 0.05, **P < 0.01, ***P < 0.001. n. s., not significant change. WT, wild‐type; Ri, RNA interference; OE, overexpression.

To further investigate how OsGRAS30 functions in OsHDAC1‐mediated immunity in response to *M. oryzae*. We first detected the abundance of OsHDAC1 proteins in WT, *OsGRAS30* mutant and *OsGRAS30* OE lines by immunoblotting using an antibody against OsHDAC1. The results indicated that the OsHDAC1 protein level remained unchanged in the *OsGRAS30* mutant and *OsGRAS30* OE lines (Figure S5a), and the transcript level of *OsHDAC1* was also unchanged in *OsGRAS30* mutant (Figure S5b,c), suggesting that OsGRAS30 did not affect OsHDAC1 abundance or stability. Thus, we speculated that OsGRAS30 interferes with the deacetylation activity of OsHDAC1. To confirm this hypothesis, we analysed HDAC activity in WT, *OsGRAS30* mutants, and OE plants, and the results showed that HDAC activity was significantly elevated in *osgras30* #1 and #2 lines and markedly reduced in *OsGRAS30* OE4 and OE8 lines compared with WT (Figure 5a). *In vitro* activity analysis revealed that OsGRAS30 inhibited the deacetylation activity of OsHDAC1 by binding to OsHDAC1 (Figure 5b).

HDACs function to remove the acetyl group from acetylated lysines in histones (Kumar *et al*., 2021). Thus, we assessed overall histone acetylation in WT, *OsHDAC1* RNAi and *OsHDAC1* OE lines by immunoblotting, and the results showed that the overall histone 3 acetylation (H3ac) level was dramatically elevated in *OsHDAC1* RNAi lines and dramatically reduced in *OsHDAC1* OE lines compared with WT (Figure 5c). Previous reports have indicated that H3K27me3 and H3K27ac regulate functional genes in *M. oryzae* (Lin *et al*., 2022; Zhang *et al*., 2021), and H3K9ac plays an essential role in plant defence responses in rice (Chen *et al*., 2022a). H3K9ac and H3K27ac are the most characterized epigenetic marks invariably associated with transcriptional activation and have been shown to affect many developmental and biological processes in higher plants (Dasgupta *et al*., 2022; Zhou *et al*., 2010). This prompts us to further assess the level of histone H3 acetylation at lysines 9 and 27 by immunoblotting using specific antibodies. The results indicated that the H3K27ac levels were higher in the *OsHDAC1* RNAi lines and lower in the *OsHDAC1* OE lines compared with WT rice plants (Figure 5c), suggesting that H3K27ac is a critical regulatory site of OsHDAC1. We then challenged rice plants with the virulent *M. oryzae* Guy11, and observed that H3K27ac levels were upregulated after *M. oryzae* infection (Figure S6). These results confirmed that H3K27ac was involved in blast defence responses in rice. Further, we detected total acetylation levels of histone 3 and 4 in *OsGRAS30* mutant lines, *OsGRAS30* OE lines, *OsHDAC1* RNAi */osgras30* #1 lines, and WT plants to assess whether OsGRAS30 impacts OsHDAC1‐mediated H3 acetylation level. The results showed that overall H3ac levels were decreased in *OsGRAS30* mutant lines, but *OsHDAC1* RNAi lines in the *osgras30 #*1 background restored this decrease, while H3ac levels were elevated in the *OsGRAS30* OE lines (Figure 5d). We specifically examined H3K9ac and H3K27ac levels in these lines and found that H3K27ac shows a similar trend to H3ac (Figure 5d). These results demonstrate that OsGRAS30 regulates H3K27ac levels by suppressing the deacetylated activity of OsHDAC1. In addition, we applied a series of different concentrations of HDAC inhibitors including trichostatin A (TSA) and sodium butyrate (NaB) to WT plants inoculated with spore suspensions for 5 days and found that the resistance of rice plants to blast infection increased with the HDAC inhibitor concentration (Figure S7). Furthermore, treatment of WT and *osgras30* #1 rice plants with HDAC inhibitors greatly improved their symptoms, and *osgras30* #1 treated with HDAC inhibitors showed greater blast resistance compared with untreated WT plants (Figure 5e,f). Taken together, these observations demonstrate that OsGRAS30 enhances blast resistance by suppressing OsHDAC1 in rice.

### OsHDAC1 and H3K27ac co‐occupy targets in rice genome

**Figure 6:**
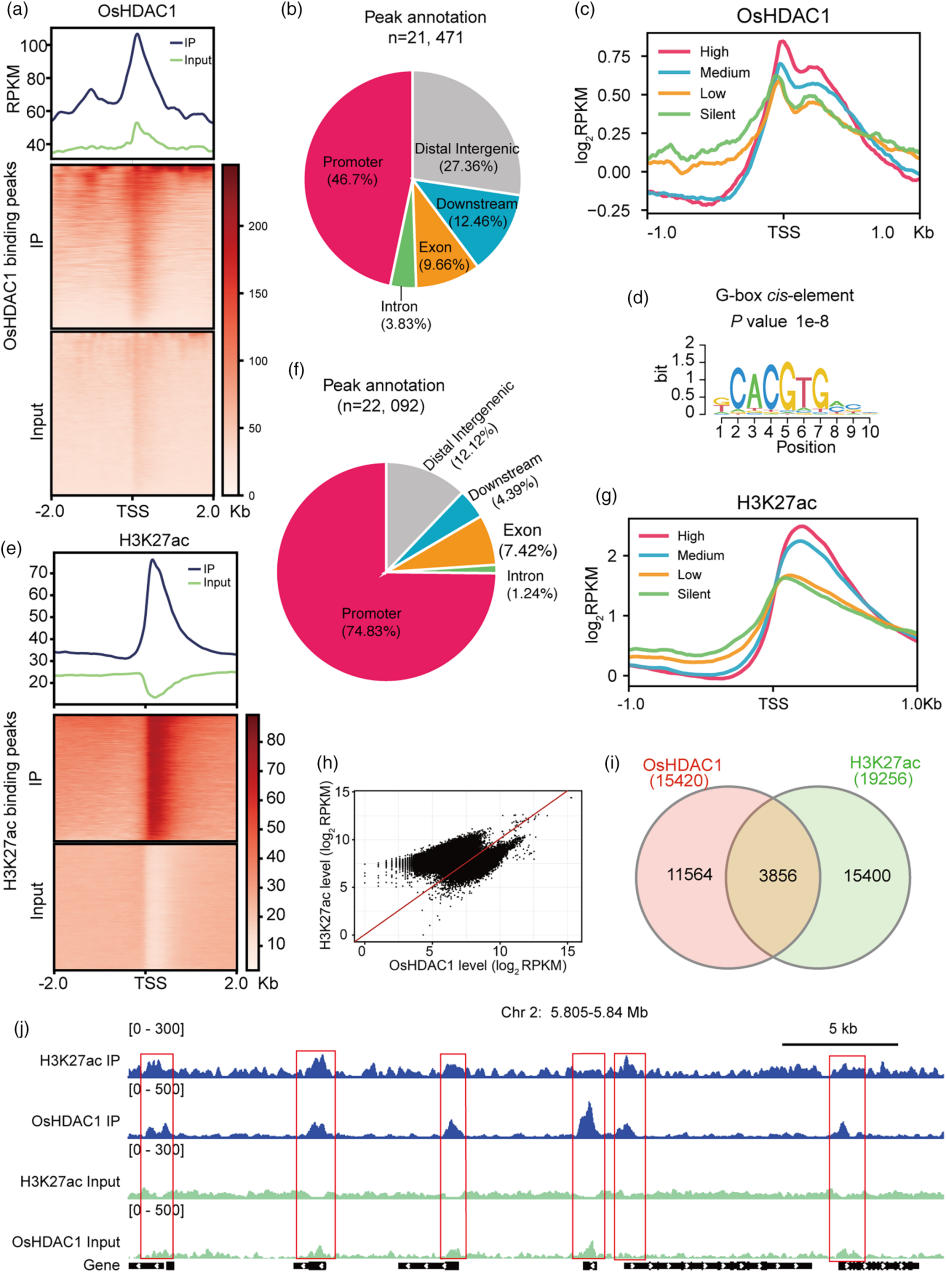
OsHDAC1 co‐occupies with H3K27ac at genome wide. (a) Metagene plot and heatmap showing OsHDAC1 binding levels on the 2.0‐kb flanking genomic region surrounding TSS in rice. (b) Pie chart showing the distribution of OsHDAC1 binding peaks at annotated genic and intergenic regions in the rice genome. (c) Metagene plot showing OsHDAC1 binding profiles on the 1.0‐kb flanking genomic region surrounding TSS of genes with high, medium, low and silent expression. (d) DREME identified representative DNA motifs in OsHDAC1 binding sites. (e) Metagene plot and heatmap showing H3K27ac enrichment levels on the 2.0‐kb flanking genomic region surrounding TSS in rice. (f) Pie chart shows the distribution of H3K27ac enrichment peaks at annotated genic and intergenic regions in the rice genome (g) Metagene plot showing OsHDAC1 binding profiles on the 1.0‐kb flanking genomic region surrounding TSS of genes with high, medium, low and silent expression. (h) Correlation between OsHDAC1 binding and H3K27ac level in OsHDAC1 target genes. (i) Venn diagram showing overlap of OsHDAC1 binding genes and H3K27 acetylated genes. (j) IGV screen shoot showing the co‐localization between OsHDAC1 and H3K27ac at a representative region in chromosome 2. TSS, the transcription start site.

To elucidate the molecular determinants and targets of OsHDAC1, we performed chromatin immunoprecipitation sequencing (ChIP–Seq) to detect OsHDAC1 binding sites in the genome of the *OsHDAC1* OE5 line using an antibody against the HA tag (Figure S8) because the HA antibody performed better than did our customized OsHDAC1 antibody in ChIP and two biological replicates showed a strong correlation (Figure S9). A total of 21, 471 OsHDAC1‐binding sites, corresponding to 15, 420 genes, were identified (Dataset S1). Analysis of the OsHDAC1 distribution in the rice genome showed that among the OsHDAC1 targets, 60.19% of binding peaks occurred in gene regions and OsHDAC1 enrichment was mostly detected near transcription start site (TSS), indicating that OsHDAC1 were highly enriched in the promoter region surrounding the TSS to regulate transcriptional initiation (Figure 6a,b). Examination of the correlation between OsHDAC1 binding and gene expression levels revealed that OsHDAC1 was more enriched in genes with high‐transcription levels than in genes with low‐transcription levels, and which was located mainly around the TSS of these genes (Figure 6c). We further identified potential DNA‐binding motifs in OsHDAC1‐binding sites using DREME, and a significantly enriched consensus G‐box motif was identified (Figure 6d), which is similar to the report that HAD9 in *Arabidopsis* could bind to the G‐box (Chen *et al*., 2016). Therefore, OsHDAC1 is associated mainly with active genes and is preferentially enriched in their promoter regions.

Next, we investigated genome‐wide targets of H3K27ac by ChIP‐Seq using an antibody against H3K27ac in WT plants with two biological replicates, whose results showed a strong correlation (Figure S10). A total of 22, 092 H3K27ac peaks, corresponding to 19, 256 genes, were identified (Dataset S2). Analysis of the H3K27ac distribution in the rice genome showed that H3K27ac peaks were located mainly in gene regions (83.49%), especially in promoter regions surrounding the TSS, and H3K27ac enrichment was mostly detected around the TSS (Figure 6e,f). In addition, we observed that H3K27ac was enriched in genes with high‐transcription levels and occurred mainly around the TSS of these genes, whereas silent genes showed a low level of H3K27ac enrichment (Figure 6g). Interestingly, analysis of the relationship between OsHDAC1 binding and acetylation levels showed that OsHDAC1 tends to be positively correlated with the H3H27ac level at the target sites (Figure 6h), and 3, 856 genes were co‐bound by OsHDAC1 and H3K27ac (Figure 6i). In humans, HDAC levels have also been shown to positively correlate with histone acetylation levels, and it is suggested that HDACs function to remove the acetyl group added by HATs in active genes and to reset chromatin modification after gene activation (Wang *et al*., 2002, 2009). Finally, a randomly selected region on chromosome 2 confirmed that OsHDAC1‐occupied peaks indeed colocalized with H3K27ac (Figure 6j), which further supports the correlation between OsHDAC1 and H3K27ac. Overall, we discovered that H3K27ac is associated with expressed genes in the rice genome and that OsHDAC1 functions to remove the acetyl moieties from H3K27ac to control the H3K27ac level in co‐occupied target genes.

### OsHDAC1 modulate blast resistance by directly suppressing blast resistance‐related genes

**Figure 7:**
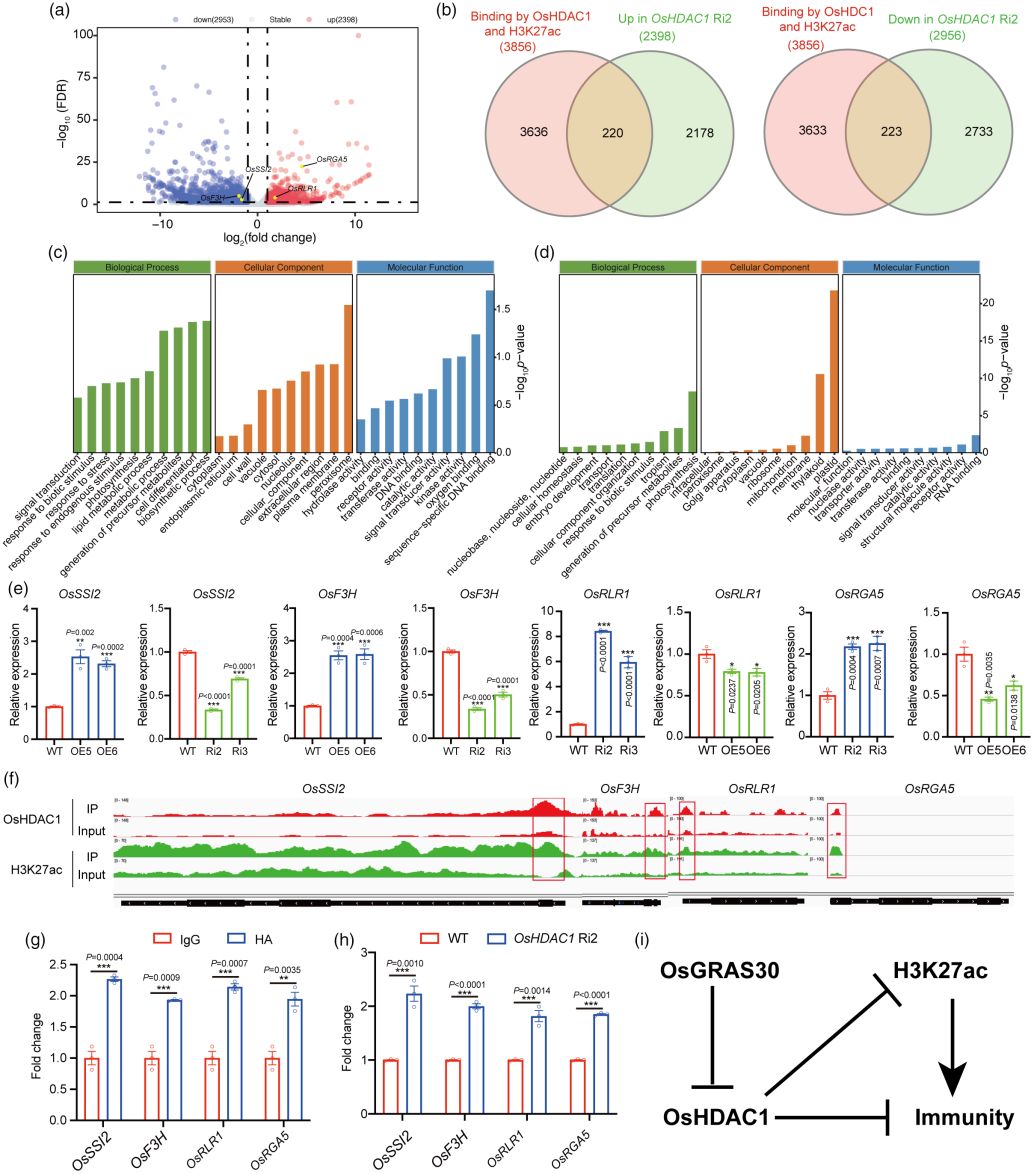
OsHDAC1 regulates the expression of blast resistance‐related genes. (a) Volcano plot showing differentially expressed genes (DEGs) associated with OsHDAC1 in the *OsHDAC1* Ri2 line compared with the WT. (b) Venn diagram showing overlap of DEGs in the *OsHDAC1* Ri2 line, OsHDAC1 binding genes and H3K27ac enrichment genes. (c) Go results showing the related biological pathway enriched from downregulated DEGs in *OsHDAC1* Ri2 line compared with WT. (d) Go results showing the related biological pathway enriched from upregulated DEGs in *OsHDAC1* Ri2 line compared with WT. (e) The transcript level of blast resistance‐related genes in *OsHDAC1* OE and RNAi lines compared with WT. Total RNA was extracted from two‐week‐old rice leaves and subjected to RT–qPCR. Expression levels of genes in the WT were set to 1.00. Values are mean ± SEM (*n* = 3). Experiments were repeated three times with similar results. (f) IGV views showing OsHDAC1 and H3K27ac enrichment in blast resistance‐related genes. Input serves as the negative control. (g) ChIP‐qPCR results of OsHDAC1 binding levels at blast resistance‐related genes in *OsHDAC1* OE plants. ChIP‐qPCR results of IgG binding levels of genes were set to 1.00. Values are mean ± SEM (*n* = 3). Experiments were repeated three times with similar results. (h) ChIP‐qPCR results of H3K27ac levels at blast resistance‐related genes in *OsHDAC1* Ri2 line compared with WT plants. ChIP‐qPCR results of H3K27ac levels of genes in the WT were set to 1.00. Values are mean ± SEM (*n* = 3). Experiments were repeated three times with similar results. Asterisks mark significant changes compared with WT (e and h) or IgG (g) based on Student's *t* test: *P < 0.05, **P < 0.01, ***P < 0.001. (i) A proposed working model showing the role of OsGRAS30 in manipulating the OsHDAC1‐mediated broad‐spectrum blast resistance in rice. WT, wild‐type; Ri, RNA interference; OE, overexpression.

To further investigate the effect of OsHDAC1 on gene expression associated with rice resistance to blast. We first profiled mRNA from 2‐week‐old rice leaves of WT and *OsHDAC1* Ri2 plants via RNA‐Seq. There are three biological replicates, whose results showed a strong correlation (Figure S11). In total, 2, 398 upregulated and 2, 953 downregulated genes (expression level fold change >2 and FDR <0.05) were identified in the *OsHDAC1* RNAi lines compared with corresponding WT plants (Figures 7a and S12; Dataset S3). Next, we investigated the association between OsHDAC1 and H3K27ac binding and altered gene expression in *OsHDAC1* Ri2 plants. The results showed that the expression of 220 genes bound by OsHDAC1 and enriched by H3K27ac was significantly increased in the *OsHDAC1* Ri2 lines, while the expression of 223 genes bound by OsHDAC1 and enriched by H3K27ac was significantly decreased in the *OsHDAC1* Ri2 lines (Figure 7b).

The differentially expressed genes (DEGs) were functionally characterized based on Gene Ontology (GO) analysis. The main GO terms assigned to downregulated DEGs affected by OsHDAC1 were the biosynthetic process and metabolic process (Figure 7c; Dataset S4). The KEGG analysis revealed that these downregulated DEGs enriched in fatty acid degradation and biosynthesis (Figure S13a; Dataset S5). Prior study revealed that fatty acid was involved in broad‐spectrum blast resistance in rice (He *et al*., 2020), suggesting that OsHDAC1 negatively regulates blast resistance by modulating fatty acid contents. Suppression of the fatty acid desaturase gene *OsSSI2* has been reported to enhance resistance to blast by regulating fatty acid content (Jiang *et al*., 2009), and we verified that OsHDAC1 could increase the expression of *OsSSI2* by binding its promoter region and altering the H3K27ac levels (Figure 7e–h). Similarly, *OsF3H* has been reported to involve in blast resistance by mediating the biosynthesis, signalling and transport of SA (Chen *et al*., 2022b; Wu *et al*., 2022). Our result showed that *OsF3H* is the direct target of OsHDAC1 by RT–qPCR and ChIP–PCR experiments (Figure 7e–h). Conversely, we found that upregulated DEGs involved in biotic stimulus were enriched (Figures 7d and S13b, Dataset S6 and S7), and genes related to blast resistance (*OsRLR1* and *OsRGA5*) were upregulated in *OsHDAC1* Ri2 plants and downregulated in *OsHDAC1* OE5 plants (Figure 7e). Among, *OsRLR1* confers broad‐spectrum resistance in rice by increasing hydrogen peroxide (H_2_O_2_) content (Du *et al*., 2021); OsRGA5 recognizes the *M. oryzae* effector AVR1‐CO39 to relieve OsRGA4 to promote blast resistance (Césari *et al*., 2013, 2014; Okuyama *et al*., 2011), supporting the role of these genes in blast resistance. Further, we found that OsHDAC1 was significantly enriched in *OsRLR1* and *OsRGA5* via ChIP–qPCR, and the H3K27ac levels in these target genes were significantly elevated in the *OsHDAC1* Ri2 lines compared with WT plants (Figure 7f–h). Together, these results suggest that OsHDAC1 binds to blast resistance‐related genes and alters their H3K27ac levels to regulate their expression, revealing a link between blast resistance and OsHDAC1.

## Discussion

Rice feeds half of the world's population, and rice blast is a very serious disease that affects rice production (Chakraborty *et al*., 2021). Functional characterization of non‐race specific resistance‐related genes is particularly important for molecular breeding to improve broad‐spectrum disease resistance and the development of strategies for effective control of this disease in rice. In this study, we report the functional identification of the OsGRAS30–OsHDAC1 module in rice blast resistance. OsGRAS30 acts genetically upstream of OsHDAC1 and interacts with OsHDAC1, repressing its deacetylation activity; inactivated OsHDAC1 induces the H3K27ac hyperacetylation and regulates the expression of blast resistance‐related genes, leading to broad‐spectrum blast resistance (Figure 7i).

### Silencing of *OHDAC1* confers broad‐spectrum resistance to *M. Oryzae* in rice

In plants, histone deacetylases are involved in the regulation of both growth and stress response functions (Chakraborty *et al*., 2021). A few HDAC members have been reported to regulate plant defence pathways, including HDA6 (Wang *et al*., 2017), HDA19 (Choi *et al*., 2012; Kim *et al*., 2008), HD2B (Latrasse *et al*., 2017) and SRT2 (Wang *et al*., 2010) in *Arabidopsis*; HDA701 (Chen *et al*., 2022a) and HDT701 (Ding *et al*., 2012; Li *et al*., 2021) in rice; and HDA6 in wheat (Zhi *et al*., 2020). Although HDA701 and HDT701 have been demonstrated to confer disease resistance in rice, the downstream factors related to their role in plant immunity remain unclear (Chen *et al*., 2022a; Li *et al*., 2021). Previous studies showed that homozygous *OsHDAC1* mutant seeds could not be obtained and that partial inhibition of *OsHDAC1* repressed root growth (Hou *et al*., 2022). In this work, the protein abundance of OsHDAC1 was downregulated at the early stage of blast infection (Figure 1b) and we further demonstrate that partial inhibition of *OsHDAC1* contributes to plant defence against multiple isolates of *M. oryzae* without affecting plant height and tiller numbers (Figures 1 and S3). Similarly, mutations in a few genes are reported to constitutively promote the nucleotide‐binding leucine‐rich repeat immune response, as well as to cause plant growth retardation (Palma *et al*., 2010; Shirano *et al*., 2002). These findings suggest that OsHDAC1 is important for rice growth but knockdown of *OsHDAC1* enhances broad‐spectrum resistance to rice blast fungus *M. oryzae*, making it a prime candidate gene for manipulation in breeding programs.

### OsGRAS30 suppresses OsHDAC1 activity, leading to enhanced blast resistance in rice

OsGRAS30 is a member of the GRAS family, and previous studies have revealed that GRAS transcription factors function in plant growth, development, and resistance to various biotic and abiotic stresses (Jaiswal *et al*., 2022). Our data suggest that OsGRAS30 positively regulates blast resistance in rice, which is supported by our finding that loss of function and overexpression of *OsGRAS30* decreased and increased blast resistance, respectively (Figure 3). Previous reports showed that EAR domain‐containing transcription factors trigger PRC2‐mediated chromatin marking in *Arabidopsis* (Baile *et al*., 2021). HDA9 and WRKY53 transcription factors are mutual antagonists (Zheng *et al*., 2020), and MADS‐domain transcription factor AGL15 involves in the recruitment of histone deacetylase complex components (Hill *et al*., 2008). Thus, the interaction of transcription factor and chromatin remodelling components is valuable for understanding the transcription factor as well as the chromatin remodelling mechanism. Our *in vitro* and *in vivo* assays both suggest that interaction between OsGRAS30 and OsHDAC1 (Figure 2), and overexpression of *OsGRAS30* led to reduced OsHDAC1 deacetylation capacity and enhanced blast resistance, while mutation of *OsGRAS30* increased blast sensitivity (Figures 3 and 5b,c), indicating that OsGRSA30 functions in blast resistance by inhibiting OsHDAC1 activity. This revealed that a GRAS transcription factor is involved in plant resistance responses by modulating the activity of an epigenetic modification enzyme. Additionally, the GRAS domain of OsGRAS30 binds to the histone deacetylase domain of OsHDAC1 (Figure 2f–h), and GRAS family DELLA proteins were found to interact with the DNA‐binding domains of EIN3/EIL1, which blocks DNA binding domain of EIN3/EIL1 to inhibit its transcriptional regulation of downstream genes in Arabidopsis (An *et al*., 2012). This implies that OsGRAS30 could block histone deacetylase domain of OsHDAC1 to inhibit its deacetylation capacity. Meanwhile, we did not detect that MBP‐OsGRAS30‐His was acetylated *in vitro*, suggesting OsGRAS30 is not a substrate for OsHDAC1. Further, the blast resistance phenotype of the *OsGRAS30* mutant lines was reversed by *OsHDAC1* RNAi (Figure 4b,c), validating that OsGRAS30 can directly target OsHDAC1 to decrease OsHDAC1 enzyme activity. HDACs function to remove the acetyl group from acetylated lysines in histones (Kumar *et al*., 2021), and we revealed that H3K27ac is a favoured substrate of OsHDAC1 (Figure 5c), while OsGRAS30 induces H3K27ac hyperacetylation by regulating the activity of OsHDAC1 (Figure 5d).

It has revealed that acetylation plays an essential role in crop defences during plant–pathogen interactions. For example, infection of maize by the fungus *Cochliobolus carbonum* increases the acetylation level of histones H3 and H4, which affects the expression of defence‐related genes (Walley *et al*., 2018). In rice, the HD2‐type HDAC HDT701 regulates plant innate immunity by modulating H4K5 acetylation in defence‐related genes (Ding *et al*., 2012), and another report revealed that OsRpp30 confers bacterial, fungal and viral resistance by targeting HDT701 to regulate the H4K5 acetylation level (Li *et al*., 2021). Similarly, the RPD3‐type HDAC HDA701 affects disease resistance by regulating H3K9 acetylation (Chen *et al*., 2022a). Our data reveal that the RPD3‐type HDAC OsHDAC1 is associated with the removal of acetyl groups from H3K27, but not H3K9 (Figure 5c), suggesting that OsHDAC1 may use a different mechanism than HDA701 to regulate blast resistance.

### Genome‐wide mapping of OsHDAC1 targets reveals its regulatory mechanism

HDACs are generally associated with transcriptional repression (Berger, 2007; Robert *et al*., 2004). However, recent genome‐wide occupancy studies in plants including *Arabidopsis* and maize (Chen *et al*., 2016; Yang *et al*., 2016), as well as humans (Wang *et al*., 2009), indicate that HDACs mainly target transcriptionally active genes. In accordance with this conclusion, our data provide evidence that the rice RPD3‐type HDAC OsHDAC1 is preferentially enriched in promoter regions and positively related to gene expression (Figure 6a–c). In addition, OsHDAC1 can bind to the G‐box (Figure 6d), which is consistent with the result obtained for HAD9 in *Arabidopsis* (Chen *et al*., 2016). These findings suggest that the interaction of HDACs with active genes may be conserved across plants and mammalian cells. Interestingly, our data show that the level of OsHDAC1 binding tends to be positively correlated with the enrichment of H3K27ac (Figure 6h,j). Similarly, the level of HDA101 binding also appears to be positively correlated with the amount of H4K5ac in maize (Yang *et al*., 2016). The probable explanation is that HDACs function to remove acetyl groups at active genes added by HATs and to reset chromatin modification after gene activation (Wang *et al*., 2009). 25.01% of OsHDAC1‐bound genes happen H3K27ac enrichment (Figure 6i), implying that OsHDAC1 bind to these target genes and controls the H3K27ac level. In addition, the more direct targets of OsHDAC1 show no H3K27ac enrichment (Figure 6i). A possible reason for this finding is that other target genes that bind to OsHDAC1 undergo acetylation at sites other than H3K27. Furthermore, our RNA‐Seq data indicated that the knockdown of *OsHDAC1* did not affect the transcript levels of most genes bound by OsHDAC1 (Figures 6i and 7b), in accordance with HDACs identified in mammalian cells, maize and *Arabidopsis* (Chen *et al*., 2016; Wang *et al*., 2009; Yang *et al*., 2016).

Plant disease resistance responses involve transcriptional reprogramming and expression changes in many genes (Tsuda and Somssich, 2015). Rice blast resistance is a complex process in which the expression of numerous genes must be fine‐tuned (Tsuda and Somssich, 2015). Blast R gene‐mediated resistance depends on interactions between R proteins and corresponding pathogenic Avr proteins (Lazar *et al*., 2022), such as *OsRGA5* positively regulates rice blast resistance by affecting the recognition of pathogenic Avr‐CO39 protein (Cesari *et al*., 2013). In addition, *OsRGA4* and *OsRGA5* are a pair of R genes for blast resistance, and both are required for blast resistance (Césari *et al*., 2013; Okuyama *et al*., 2011), which upon recognition of the pathogen effector AVR‐Pia by direct binding to OsRGA5, OsRGA4 is relieved to induce cell death (Césari *et al*., 2014). Upregulation of *OsRGA5* due to *OsHDAC1* knockdown may possibly contribute to the recognition of the *M. oryzae* effectors. *OsRLR1* improve broad‐spectrum blast resistance by regulating H_2_O_2_ accumulation (Du *et al*., 2021). *OsRGA5*, along with *OsRLR1* were identified downstream of OsHDAC1. Our results indicate that OsHDAC1 bind to the promoter region of these genes and alters the H3K27ac levels, inhibiting their expression to suppress immune activation (Figure 7). Thus, an important finding of our study is that positive regulators of blast resistance‐related genes are associated with OsHDAC1 binding and are inhibited by OsHDAC1 (Figure 7). Conversely, negative regulators of blast resistance‐related genes associated with OsHDAC1 binding are upregulated by OsHDAC1 (Figure 7). These genes including *OsF3H* and *OsSSI2* caused the decreased production of SA and fatty acid, and their inhibition could achieve broad‐spectrum resistance to *M. oryzae* in rice (Chen *et al*., 2022b; Jiang *et al*., 2009). Overall, our findings revealed that OsHDAC1 directly affect these targets and thus alters their expression to regulate blast resistance in rice.

### A chemical intervention strategy reduces HDAC activity to increase blast resistance in rice

Rice blast, caused by *M. oryzae*, is an important disease affecting rice production, and thus it threatens global food security (Chakraborty *et al*., 2021). The symptoms of rice blast can occur throughout the rice growth period (Chakraborty *et al*., 2021). Genetic breeding of durably resistant plants is considered an effective strategy for controlling plant diseases, although it is difficult and time consuming to accomplish without affecting plant growth due to the limited set of currently available broad‐spectrum resistance genes without detrimental effects (He *et al*., 2022). Some genes have been engineered to increase disease resistance directly. The present study showed that knockdown of *OsHDAC1* via RNAi conferred rice blast disease resistance, and our preliminary field test results showed no difference in plant height and tiller numbers, between *OsHDAC1*‐knockdown and WT rice (Figures 1 and S3), revealing that the development of resistant rice can be achieved by decreasing the expression, rather than inducing loss of function, of a target gene. Another strategy for disease resistance is chemical intervention, i.e., application of small‐molecule chemicals or drugs to enhance resistance via interference with the functions of target genes. HDAC inhibitor treatment enhanced rice blast resistance (Figure S7) provides a foundation for further application of such chemicals to disease intervention *in vitro*.

## Materials and methods

### Plasmid construction and generation of transgenic plants

For the purification of OsHDAC1‐His and MBP‐OsGRAS30‐His recombinant proteins, a fragment containing the *OsHDAC1* coding sequence (CDS) was amplified and cloned into the *EcoR*I site of the pET32a vector, and the *OsGRAS30* CDS fragment was amplified and inserted into the *EcoR*I site of the pMalC2x vector. To generate gene constructs fused to the DNA binding domain or activation fragment for Y2H assays, the *OsHDAC1* CDS fragments, as well as the N‐terminal region (nucleotides 1–63), histone deacetylase domain region (nucleotides 64–1197), and C‐terminal region (nucleotides 1198–1557) of *OsHDAC1* were amplified and cloned into the *EcoR*I site of the pGBKT7 vector; the *OsGRAS30* CDS fragments, as well as the N‐terminal region (nucleotides 1–255), GRAS domain region (nucleotides 256–1344) and C‐terminal region (nucleotides 1345–1503) of *OsGRAS30* were amplified and cloned into the *EcoR*I site of the pGADT7 vector. To generate the OsHDAC1‐nLUC and cLUC‐OsGRAS30 constructs, the *OsHDAC1* CDS fragments were amplified and cloned into the *Kpn*I and *Sac*I sites of the pCambia1300‐nLUC vector, while *OsGRAS30* CDS fragments were amplified and cloned into the *Kpn*I and *Sal*I sites of the pCambia1300‐cLUC vector. To generate the OsGRAS30‐mCherry constructs, the amplified *OsGRAS30* CDS fragments were fused to a mCherry tag to generate fusion genes and cloned into the *BamH*1 and *Sal*I sites of the pBI121 vector. To generate *OsGRAS30* OE plants, the amplified *OsGRAS30* CDS fragment was fused to a MYC tag to generate fusion genes and cloned into the *Xcm*I site of the pCXUN vector. To generate *OsGRAS30* mutant plants, guide sequences were designed using CRISPR‐P (http://cbi.hzau.edu.cn/crispr) and inserted into the sgRNA expression vector backbone pYLsgRNA‐OsU6 using annealed oligonucleotides. Then, the sgRNAs were inserted into the pYLCRISPR/Cas9Pubi‐H vector as described previously (Hou *et al*., 2022). The OsGRAS30‐MYC and CRISPR/Cas9‐OsGRAS30 constructs were transfected into *A. tumefaciens* strain EHA105 for transformation of WT (Nipponbare) and OsHDAC1‐RNAi constructs (Hou *et al*., 2022) were transfected into EHA105 for transformation in the background of *osgras30* #1. These transformations were performed by Wuhan BioRun Biosciences Co., Ltd. (Wuhan, China).

### Plant materials and growth conditions

All rice plants used in this study, including Nipponbare (*Oryza sativa* L. *ssp. japonica*), the *OsHDAC1* RNAi and OE lines in the background of Nipponbare (Hou *et al*., 2022), the *OsGRAS30* mutants and OE lines, and the *OsHDAC1* RNAi lines in the background of *osgras30* #1 were grown in hydroponic cultures in Kimura B nutrient salts under a photoperiod of 14 h light at 28 °C and 10 h dark at 26 °C with 70% humidity. For growth in the field, germinated rice seedlings were transplanted into the Huashan rice experimental paddy fields at the Wuhan University in Wuhan, Hubei province, China in mid‐May; grains were harvested in October. Urea was used as the nitrogen source, applied at 2 kg N/100 m^2^. The rice plants were transplanted at a density of three rows of 38 plants with spacing between rice plants of 20 cm. The *N. benthamiana* plants used in this study were grown in soil under a photoperiod of 14 h light at 22 °C and 10 h dark at 22 °C with 70% humidity.

### Inoculation of rice leaves with *M. Oryzae* strains

The *M. oryzae* strains Guy11, AH4, Sc09‐153‐07, TM3‐2, RB1, RB3, NC1, HLJ1‐3, HLJ09‐17‐1 and HLJ5‐3 were used in this study. *M. oryzae* cultivation and inoculation assay were performed using methods described previously with modifications (Li *et al*., 2017). Briefly, *M. oryzae* strains were gown on complete agar medium in a growth chamber at 25 °C under a 12‐h light/12‐h dark photoperiod to produce spores, which were collected with sterile water at a final concentration of 5 × 10^5^ spores/mL in suspension. For spray inoculation, 2‐week‐old rice seedlings were sprayed in a dew growth chamber with spore suspensions from various *M. oryzae* strains. At 5 days after inoculation, the lesion area was measured using Photoshop according to a previously reported method (Li *et al*., 2017), and relative fungal biomass was determined by qPCR analysis of the *M. oryzae POT2* gene, the expression of which was normalized to that of the rice *Ubiquitin* gene, as described previously (Wang *et al*., 2018). All inoculation experiments were repeated three times independently.

### HDAC inhibitor treatments

TSA (Selleck Chemicals, Cat. # S1045, Houston, TX, USA) was dissolved in dimethyl sulfoxide. NaB (Beyotime, Cat. # ST1636, Shanghai, China) was dissolved in double‐distilled water. Two‐week‐old rice seedlings were spray‐inoculated with *M. oryzae* spore suspensions and transferred to Kimura B nutrient salts containing the indicated concentration of TSA or NaB for 5 days. Control plants were grown in an identical volume of dimethyl sulfoxide or double‐distilled water as a mock treatment.

### RNA extraction and RT–qPCR

RNA was extracted from the leaves of 2‐week‐old seedlings with Trizol reagent (ThermoFisher, Cat. # 15596026, Waltham, MA, USA) and then reverse‐transcribed into cDNA using the SuperScript III transcriptase kit (ThermoFisher, Cat. # 18080093). RT–qPCR analysis was conducted using SYBR green master mix (Bio‐Rad, Cat. # 1725150, Hercules, CA, USA) on the CFX96 real‐time detection system 690 (Bio‐Rad, USA). The rice *Ubiquitin* (*LOC_Os03g13170*) gene was used as an internal control (Wang *et al*., 2018). The mRNA levels relative to the *Ubiquitin* level were calculated according to the 2^−∆∆Ct^ method.

### *In vitro* purification of recombinant proteins

The GST‐OsHDAC1, OsHDAC1‐His and MBP‐OsGRAS30‐His proteins were purified as described previously (Hou *et al*., 2022). These constructs were transformed into *E. coli* strain BL21(DE3); expression was induced by adding 0.5 mM isopropyl ß‐D‐1‐thiogalactopyranoside (Coolaber, Cat. # CI6621, Beijing, China) to the culture, followed by incubation for 12 h at 16 °C. The fusion proteins were purified using glutathione resin (GeneScript, Cat. # L00206, Nanjing, China) or high‐affinity Ni‐NTA resin (GeneScript, Cat. # L00250) in accordance with the manufacturer's instructions.

### Antibodies

To generate an antibody against rice OsHDAC1, purified OsHDAC1‐His recombinant protein was used as an antigen to immunize rabbits. Immunization of rabbits and serum collection were performed by Dai‐An Biotech (Wuhan, China). The antibodies used for immunoblotting and ChIP were as follows: anti‐OsHDAC1 (1:1000; Dai‐An), anti‐HA (1:5000 for immunoblot, 1:100 for ChIP; Abcam, Cat. # ab9110, Cambridgeshire, UK), anti‐MYC (1:5000; Proteintech, Cat. # 60003‐2‐Ig) anti‐IgG (1:100; Abcam, Cat. # ab172730), anti‐H3 (1:2000; Abcam, Cat. # ab1791), anti‐H3ac (1:2000; Millipore, Cat. # 17‐625, MA, USA), anti‐H4ac (1:2000; PTMbio, Cat. # PTM‐189, Hangzhou, China), anti‐H3K9ac (1:2000; Millipore, Cat. # 07–352), anti‐H3K27ac (1:5000 for immunoblot, 1:100 for ChIP; Abcam, Cat. # ab4729), anti‐His (1:5000, Proteintech, Cat. # 1B7G5, Wuhan, China), anti‐actin (1:2000, ABclonal, Cat. # AC009, Wuhan, China), goat anti‐rabbit IgG‐horseradish peroxidase (HRP) (1:5000; Beyotime, Cat. # A0277) and goat anti‐mouse IgG‐HRP (1:5000; Beyotime, Cat. # A0216).

### Protein extraction and immunoblotting

The leaves of 2‐week‐old seedlings were ground into powder in liquid nitrogen. Then, histones were extracted using a protein extraction kit (Bestbio, Cat. # BB‐31171, Shanghai, China), and total protein was extracted using another protein extraction kit (Bestbio, Cat. # BB‐319815). Protein concentrations were measured using a bicinchoninic acid protein assay kit (Bio‐sharp, Cat. # BL521A, Hefei, China). Subsequently, proteins were heated for 10 min at 95 °C, separated using a 15% or 12% sodium dodecyl sulfate–polyacrylamide electrophoresis gel, transferred to polyvinylidene fluoride membranes, and detected as described previously (Hou *et al*., 2017). All western blots were developed using the ECL Plus immunoblotting detection system (GE Healthcare, Little Chalfont, UK).

### Y2H assay

Y2H assays were performed using the Matchmaker GAL4 Two‐hybrid System 3 (Clontech, Mountain View, CA, USA) according to the manufacturer's instructions. Briefly, the bait and prey constructed in this work were co‐transformed into *S. cerevisiae* AH109 cells. After cultivation on SD medium lacking Leu and Trp (Takara, Cat. # 630316, Kyoto, Japan) at 30 °C for 3 days, yeast transformants were transferred onto SD medium lacking Leu, Trp, His and Ade (Takara, Cat. # 630322) or medium supplemented with 5‐bromo‐4‐chloro‐3‐indoxyl‐α‐d‐galactopyranoside (Takara, Cat. # 630463) and grown at 30 °C for 3 days.

### Co‐IP assay

Co‐IP assays were performed as described previously (Zhang *et al*., 2017). The OsHDAC1‐HA and OsGRAS30‐MYC constructs or HA and OsGRAS30‐MYC constructs were transformed into rice protoplasts using a plant protoplast preparation and transformation kit (Real‐Times, Cat. # RTU4052, Beijing, China) in accordance with the manufacturer's instructions. Then, total proteins were extracted from rice protoplasts and incubated with rProtein A sepharose (GE Healthcare, USA) and an anti‐HA antibody. Proteins bound to sepharose were detected using an anti‐MYC antibody.

### Split‐LUC assay

Split‐LUC assays were performed according to a previously described method (Hou *et al*., 2022). The OsHDAC1‐nLUC and cLUC‐OsGRAS30 constructs were transformed into *A. tumefaciens* strain GV3101(pSoup‐p19). Equal amounts of bacterial suspension were infiltrated into the leaves of 5‐week‐old *N. benthamiana* plants. The inoculated leaves were sprayed with 0.5 mM luciferin (Coolaber, Cat. # CL6928) after 3 days and then kept in the dark for 5 min. Luminescence images were taken using the Tanon 5200 imaging apparatus (Shanghai, China).

### *In vitro* pull‐down assay

Pull‐down assays were performed as described previously (Hou *et al*., 2022). Glutathione resin containing GST or GST‐OsHDAC1 was incubated with MBP‐OsGRAS30‐His in phosphate‐buffered saline at 4 °C for 2 h. The proteins eluted from the resin were detected by immunoblotting using an anti‐His antibody.

### Subcellular localization assay

To assess the co‐localization of OsHDAC1 with OsGRAS30 in rice protoplasts, the OsHDAC1‐sGFP (Hou *et al*., 2022) and OsGRAS30‐mCherry constructs were transformed into rice protoplasts using a plant protoplast preparation and transformation kit in accordance with the manufacturer's instructions. The transformed protoplasts were observed using a confocal laser scanning microscope (Leica SP8, Wetzlar, Germany). Images were analysed using LAS‐AF software. The settings used for confocal microscopy were as follows: for sGFP, excitation 488 nm, emission 500–550 nm; for mCherry, excitation 552 nm, emission 575–625 nm; fluorescence intensity, 1%–10%; gain value of the HyD detector, 100–200.

### HDAC activity assay

To assess HDAC activity *in vivo*, leaves of 2‐week‐old seedlings from WT, *OsGRAS30* mutant and OE lines were ground into powder in liquid nitrogen and extracted using a protein extraction kit (Bestbio, Cat. # BB‐319815). Protein concentrations in each sample were measured using a bicinchoninic acid protein assay kit (Bio‐sharp, Cat. # BL521A), and approximately 4 μg total protein was used to assess *in vivo* HDAC activity. For the *in vitro* HDAC activity assay, purified recombinant GST‐OsHDAC1 (~2 μg) and MBP‐OsGRAS30‐His (~2 μg) or MBP‐His (~2 μg) proteins were incubated at 37 °C for 1 h, and the mixed proteins were used to assess *in vitro* HDAC activity. Subsequently, the mixed proteins were subjected to *in vivo* and *in vitro* HDAC activity assays using the Epigenase HDAC activity/inhibition direct assay kit (Epigentek, Cat. # P‐4034, Farmingdale, NY, USA) in accordance with the manufacturer's instructions. After the reactions were complete, the optical density at 450 nm was measured using a SpectraMax iD5 microplate reader (Molecular Devices, Sunnyvale, CA, USA). The activity of the HDAC enzyme in proportion to optical density (measured as intensity in relative fluorescence units [RFU]) was calculated using the formula:

$$\text{HDAC activity}\left(\mathrm{RFU}/\min/\mathrm{mg}\right)=\begin{array}{l} \frac{\text{Sample}\;\mathrm{RFU}-\text{Blank}\;\mathrm{RFU}}{\text{Protein amount}\left(\mathrm{\mu g}\right)*\times \min*}\\ \times 1000. \end{array}$$

### ChIP‐Seq or ‐PCR

Two‐week‐old seedlings were ground into a fine powder and suspended in a nuclear isolation buffer (10 mM HEPES [pH 8.0], 1 M sucrose, 5 mM KCl, 5 mM MgCl_2_, 0.6% Triton X‐100, 0.4 mM phenylmethylsulfonyl fluoride, 1× protease inhibitor cocktail, 1% formaldehyde) for 20 min for crosslinking. ChIP assays were performed using a ChIP kit (Bersinbio, Cat. # Bes5002, Guangzhou, China) in accordance with the manufacturer's instructions. Finally, the eluted DNA samples were digested with proteinase K (Invitrogen, Cat. # 25530049, Waltham, MA, USA) and RNase A and purified for subsequent sequencing or qPCR.

ChIP‐Seq for analysis of OsHDAC1 occupancy was performed using an antibody against the HA tag in *OsHDAC1* OE lines, with two replicates. The input was used as a negative control, and IgG was used as an experimental control for the HA antibody. ChIP‐Seq for genome‐wide H3K27ac analysis was performed using an antibody against H3K27ac in WT plants, with two replicates. Immunoprecipitated and purified DNA was used for library construction and sequencing, which were performed on the Illumina Novaseq 6000 sequencing system by Seqhealth Biotech (Wuhan, China).

ChIP–qPCR was performed as described previously (Ke *et al*., 2020). In brief, the immunoprecipitated and purified DNA was subjected to qPCR to amplify the target sequences. The *Ubiquitin* (*LOC_Os03g13170*) promoter was used as an internal reference control.

### Analysis of ChIP‐Seq data

A total of 10 Gb high‐quality 150‐bp paired‐end reads were generated from each sample. Raw data obtained from Illumina sequencing were processed and filtered using FastP to generate clean reads. These reads were mapped to the Nipponbare genome (MSU 7.0) using Bowtie2 with the default parameters, and only unique alignments were retained. The SAM‐formatted output files generated by Bowtie2 were transformed to BAM format, followed by sorting and indexing using Samtools. Peaks were called using MACS2, and all duplicate reads were retained for OsHDAC1 and H3K27ac ChIP‐Seq analysis. The q‐value cutoff applied to calculate statistical significance was <0.01. Other parameters were set to their default values. The abundance of OsHDAC1 and H3K27ac ChIP‐Seq reads was normalized to the corresponding input level. The genomic regions enriched in OsHDAC1 and H3K27ac were determined by comparison of the corresponding ChIP library with its input DNA library. All statistical analyses and figure construction were conducted using R.

### RNA‐Seq and data analysis

Total RNA was isolated from 2‐week‐old seedlings of WT and *OsHDAC1* Ri2 plants in three biological replicates using a TRIzol kit according to previously reported methods (Hou *et al*., 2021). Subsequently, mRNA enrichment, cDNA synthesis, library construction and sequencing were performed using the Illumina Novaseq 6000 system by Seqhealth Biotech (Wuhan, China).

Finally, 6 Gb high‐quality 150‐bp paired‐end reads were generated from each sample. Raw data obtained from Illumina sequencing were processed and filtered using Fastp to generate clean reads. These reads were aligned to the Nipponbare reference genome (MSU 7.0) using the splice junction mapper TopHat2. HTSeq was used to obtain the read counts mapped to each gene. The read counts were normalized as reads per kilobase per million reads to assess relative expression levels. EdgeR was used for differential expression analysis. Genes showing an absolute value of log2 fold change (*OsHDAC1* Ri2/WT) > 1 and adjusted *P*‐value <0.05 were considered differentially expressed. All the statistical analyses and figure construction were conducted using R.

### Statistical analysis

All the values are presented as the mean ± standard error of the mean, and the number (*n*) of samples is indicated. Grey values were determined using ImageJ software. Statistically significant differences between control and experimental groups were determined using Student's *t‐*test, with values of *P* < 0.05 indicating statistical significance.

### Primer sequences

The sequences of primers used in this study are listed in Table S1.

## Conflict of interest

The authors declare no competing interests.

## Author contributions

J.H. and L.L. conceived the ideas and designed the experiments. J.H. performed most experiments and data analysis. H.X., and Q.S. performed part experiments, including LUC, Pull‐down, RT–qPCR and material identification. P.Y., performed bioinformatics analysis, J.Y., and X.M. helped to perform part experiments, including Y2H, and agronomic traits analysis. H.H. performed part data analysis and discussion. J.H. and L.L. supervised the research and wrote the article.

## Supporting information


**Figure S1** Specificity testing results for the anti‐OsHDAC1 polyclonal antibody in immunoblotting analysis in WT and *OsHDAC1* OE5 plants.
**Figure S2** The standard *M. oryzae* strain Guy11 and *M. oryzae* strains AH4, Sc09‐153‐07, TM3‐2, RB1, RB3, NC1, HLJ1‐3, HLJ09‐17‐1 and HLJ5‐3 isolated in various regions of China were used for rice leaf infection analyses.
**Figure S3** Phenotypes and statistics of key agronomic traits of *OsHDAC1* RNAi and OE plants in the field.
**Figure S4** Diagrammatic representation of vector construction and expression analysis of *OsGRAS30* in transgenic plants.
**Figure S5** Protein abundance and transcript levels of *OsHDAC1* in *OsGRAS30* overexpression lines, mutants and *osgras30* #1/*OsHDAC1* RNAi lines.
**Figure S6** H3K27ac level was increased in response to *M. oryzae* Guy11 treatment, as shown by immunoblotting analysis.
**Figure S7** Rice blast disease symptoms after treatment with HDAC inhibitors.
**Figure S8** Specificity testing of the anti‐HA antibody for chromatin immunoprecipitation sequencing (ChIP‐Seq) analysis in OsHDAC1 OE5 plants.
**Figure S9** The correlation analysis of the two biological replicates of OsHDAC1 in ChIP‐seq.
**Figure S10** The correlation analysis of the two biological replicates of H3K27ac in ChIP‐seq.
**Figure S11** The correlation analysis of the three biological replicates of OsHDAC1 in RNA‐seq.
**Figure S12** Heatmap showing different expression of numerous DEGs in rice seedlings between the *OsHDAC1* Ri2 line and WT.
**Figure S13** Significantly enriched KEGG terms among OsHDAC1‐regulated DEGs.
**Table S1** Primers used in this study.

**Dataset S1** OsHDAC1 binding sites identified by ChIP‐Seq.

**Dataset S2** H3K27 distribution sites identified by ChIP‐Seq.

**Dataset S3** List of DEGS in *OsHDAC1* Ri2 plants vs WT plants.

**Dataset S4** GO pathways of downregulated DEGS in *OsHDAC1* Ri2 plants vs WT plants.

**Dataset S5** KEGG pathways of downregulated DEGS in *OsHDAC1* Ri2 plants vs WT plants.

**Dataset S6** GO pathways of upregulated DEGS in *OsHDAC1* Ri2 plants vs WT plants.

**Dataset S7** KEGG pathways of upregulated DEGS in *OsHDAC1* Ri2 plants vs WT plants.

## Data Availability

RNA‐ and ChIP‐sequencing raw data are available at the National Genomics Data Center, Beijing Institute of Genomics (China National Center for Bioinformation), Chinese Academy of Sciences, under the Genome Sequence Archive (GSA) accession number CRA012210. All data supporting the findings of this study are available within the paper and within its supplemental data.
