# Supplementary material for: Unveiling the mechanism of broad‐spectrum blast resistance in rice: The collaborative role of transcription factor OsGRAS30 and histone deacetylase OsHDAC1
Source: Plant Biotechnol J. 2024 Jan 31;22(6):1740–56. doi: 10.1111/pbi.14299 (PMC11123394; doi:10.1111/pbi.14299)
Supplement: Supplementary file 1 — Figure S1 Specificity testing results for the anti‐OsHDAC1 polyclonal antibody in immunoblotting analysis in WT and OsHDAC1 OE5 plants. Figure S2 The standard M. oryzae strain Guy11 and M. oryzae strains AH4, Sc09‐153‐07, TM3‐2, RB1, RB3, NC1, HLJ1‐3, HLJ09‐17‐1 and HLJ5‐3 isolated in various regions of China were used for rice leaf infection analyses. Figure S3 Phenotypes and statistics of key agronomic traits of OsHDAC1 RNAi and OE plants in the field. Figure S4 Diagrammatic representation of vector construction and expression analysis of OsGRAS30 in transgenic plants. Figure S5 Protein abundance and transcript levels of OsHDAC1 in OsGRAS30 overexpression lines, mutants and osgras30 #1/OsHDAC1 RNAi lines. Figure S6 H3K27ac level was increased in response to M. oryzae Guy11 treatment, as shown by immunoblotting analysis. Figure S7 Rice blast disease symptoms after treatment with HDAC inhibitors. Figure S8 Specificity testing of the anti‐HA antibody for chromatin immunoprecipitation sequencing (ChIP‐Seq) analysis in OsHDAC1 OE5 plants. Figure S9 The correlation analysis of the two biological replicates of OsHDAC1 in ChIP‐seq. Figure S10 The correlation analysis of the two biological replicates of H3K27ac in ChIP‐seq. Figure S11 The correlation analysis of the three biological replicates of OsHDAC1 in RNA‐seq. Figure S12 Heatmap showing different expression of numerous DEGs in rice seedlings between the OsHDAC1 Ri2 line and WT. Figure S13 Significantly enriched KEGG terms among OsHDAC1‐regulated DEGs. Table S1 Primers used in this study. [file PBI-22-1740-s005.docx]

**
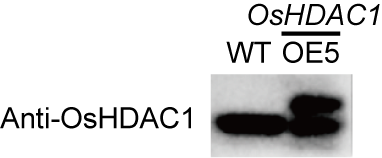
**

**Figure S1.** **Specificity testing results for the anti-OsHDAC1 polyclonal antibody in immunoblotting analysis in WT and *OsHDAC1* OE5 plants.**

Molecular weights of proteins (kDa) are shown on the left. WT, wild-type; OE, overexpression.

**
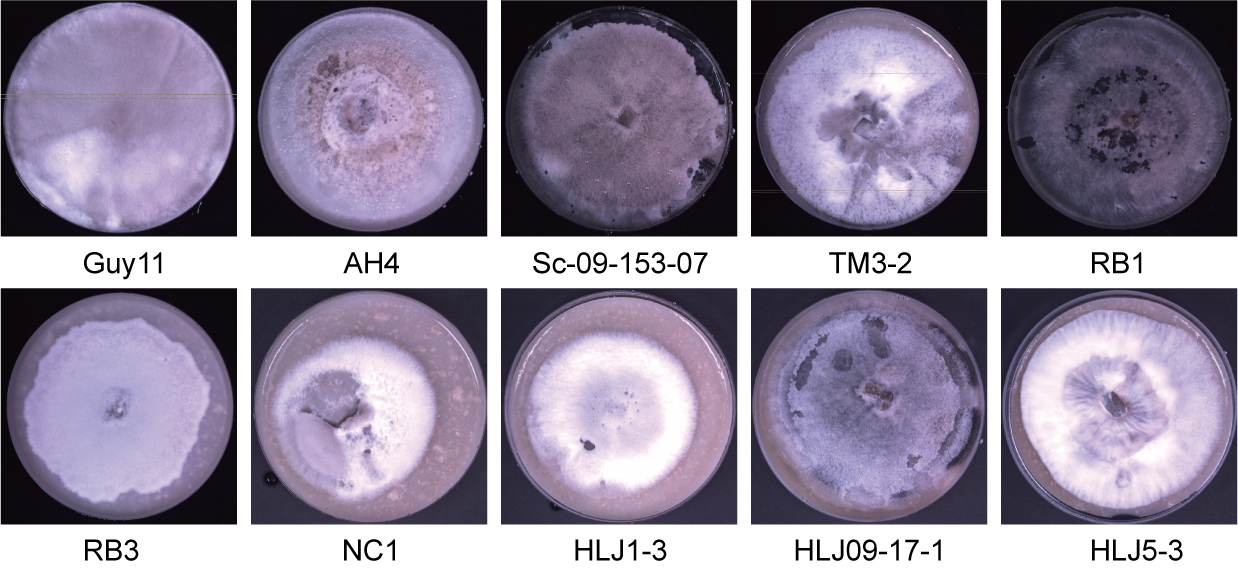
**

**Figure S2. The standard *M. oryzae* strain Guy11 and *M. oryzae* strains AH4, Sc09-153-07, TM3-2, RB1, RB3, NC1, HLJ1-3, HLJ09-17-1, and HLJ5-3 isolated in various regions of China were used for rice leaf infection analyses.**

**
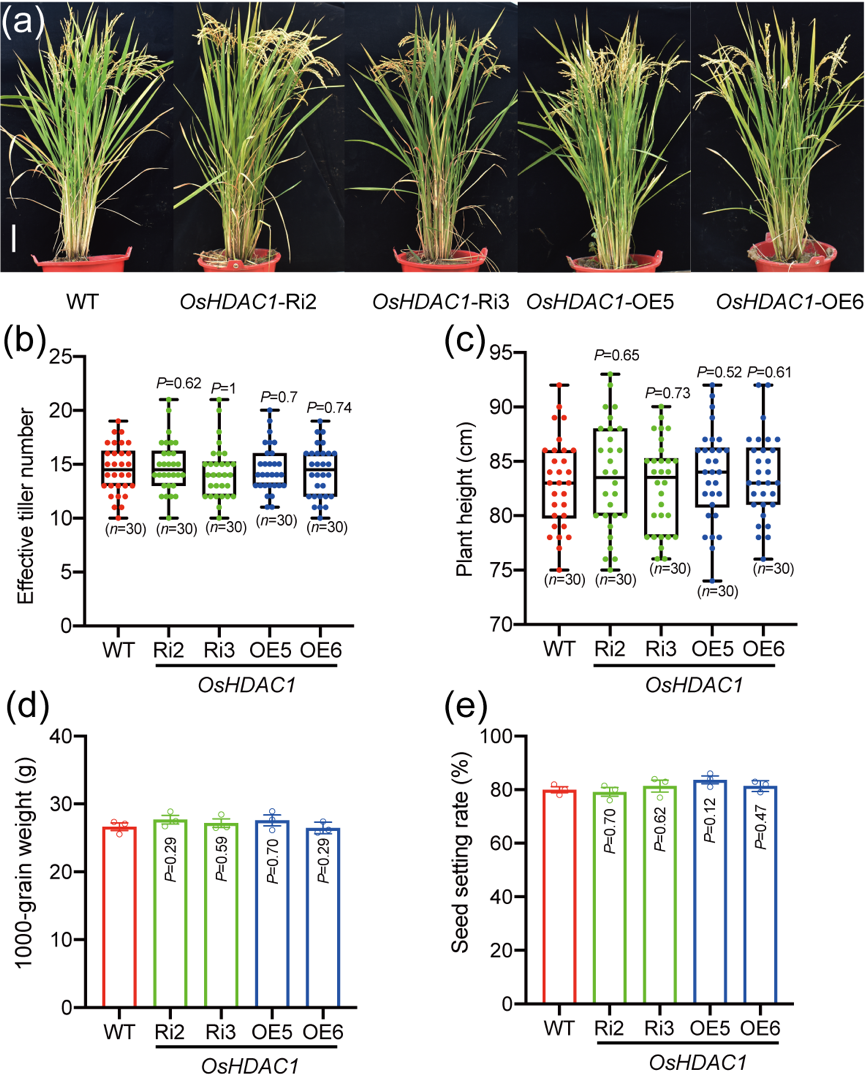
**

**Figure S3.** **Phenotypes and** **statistics of key agronomic traits of *OsHDAC1* RNAi and OE plants in the field.**

(a) Representative phenotype of *OsHDAC1* RNAi and OE plants in the field. Scale bar = 10 cm. (b-e) Comparison of four key traits: effective tiller number (b), plant height (c), 1000-grain weight (d) and seed setting rate (e) in WT and *OsHDAC1* RNAi and OE lines. Data in (b) and (c) are displayed as box and whisker plots with individual data point, the error bars represent maximum and minimum values. Center line, median (*n* = 30). Data in (d) and (e) are means ± SEM (*n* = 3). Statistical significance was determined by two-tailed Student’s *t* test.


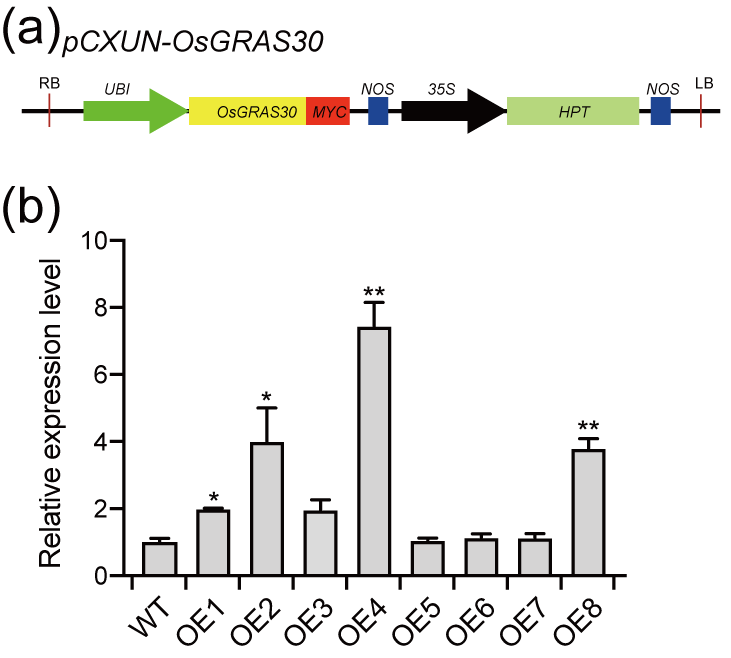


**Figure S4. Diagrammatic representation of vector construction and expression analysis of *OsGRAS30* in transgenic plants.**

(a) Schematic diagram of the plant expression vector pCXUN-OsGRAS30 used for *OsGRAS30* overexpression in transgenic plants. RB, right border; NOS, terminator of nopaline synthase gene; HPT, hygromycin resistance gene; 35S, CaMV 35S promoter; MYC, MYC epitope tag sequence; Ubi, *ubiquitin* promoter derived from *Zea mays*; LB, left border. (b) Expression level of *OsGRAS30* in the WT and *OsGRAS30* OE lines. Total RNA was extracted from two-week-old rice leaves and subjected to RT-qPCR. WT, wild-type; OE, overexpression. Values are means ± SD (*n*=3). Experiments were repeated three times with similar results. Asterisks mark significant change compared with WT based on Student’s *t* test: **P* < 0.05, ***P* < 0.01.

**
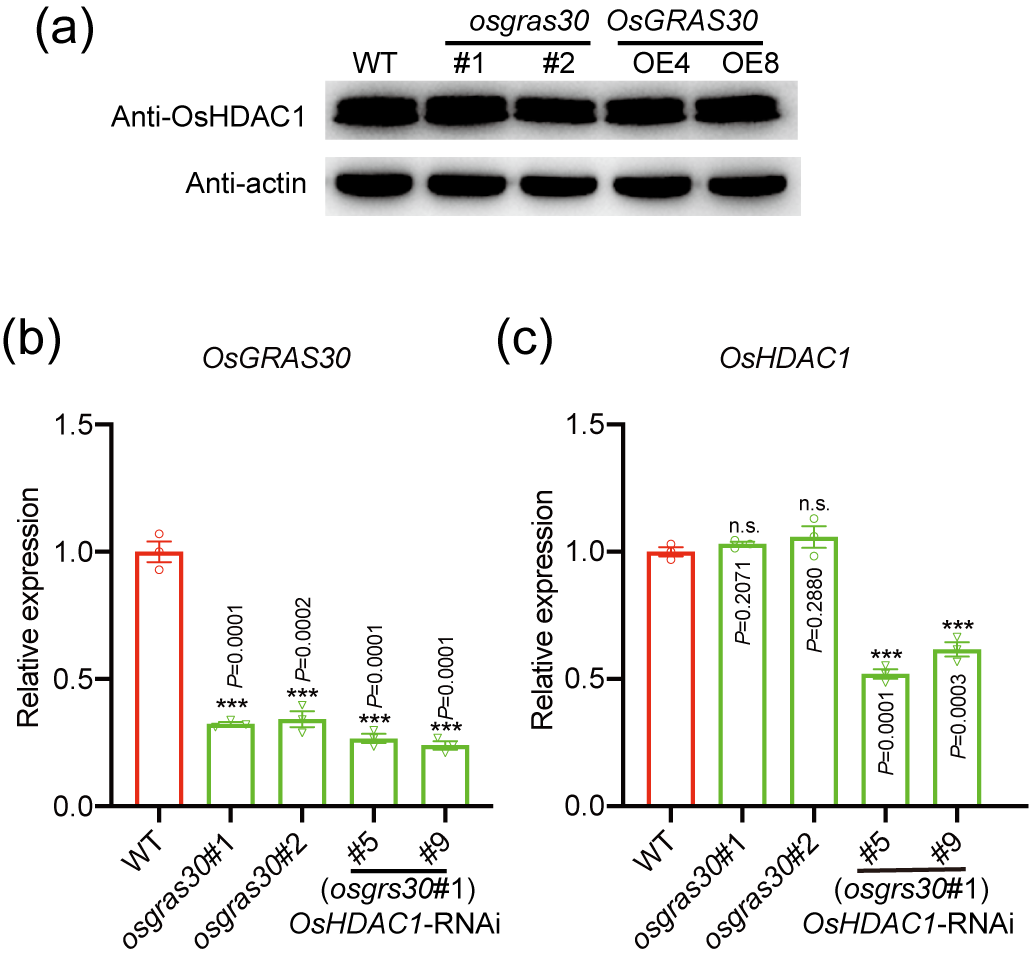
Figure S5.** **Protein abundance and transcript levels of *OsHDAC1* in *OsGRAS30* overexpression lines, mutants and *osgras30* #1/*OsHDAC1* RNAi lines.**

(a) Immunoblotting analysis of the OsHDAC1 protein levels in WT, *OsGRAS30* mutant and OE lines. Actin protein was applied as an equal loading control. (b and c) Transcript level of *OsGRAS30* and *OsHDAC1* in *OsGRAS30* mutants and *OsHDAC1* RNAi/*osgras30* #1 compared with WT plants. The transcript level of *OsGRAS30* in these lines was used as a positive control. The expression levels of genes in the WT were set to 1.00 as a control. Total RNA was extracted from two-week-old rice leaves and subjected to RT-qPCR. The expression levels of genes in the WT were set to 1.00. Values are mean ± SEM (*n* = 3). Experiments were repeated three times with similar results. Asterisks mark significant changes compared with WT based on Student’s *t* test: ****P*<0.001. WT, wild-type; RNAi, RNA interference.

**
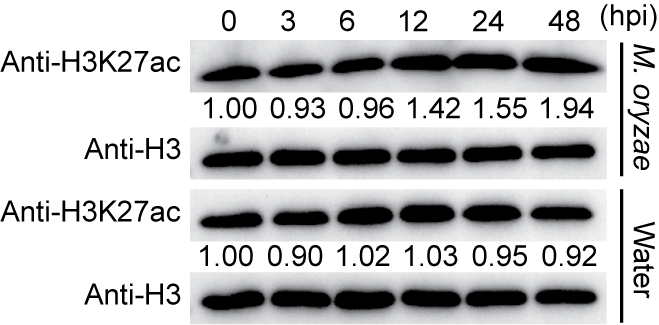
**

**Figure S6. H3K27ac level was increased in response to *M. oryzae* Guy11 treatment, as shown by immunoblotting analysis.**

Water treatment was used as a control, and the H3 protein was applied as an equal loading control.

**
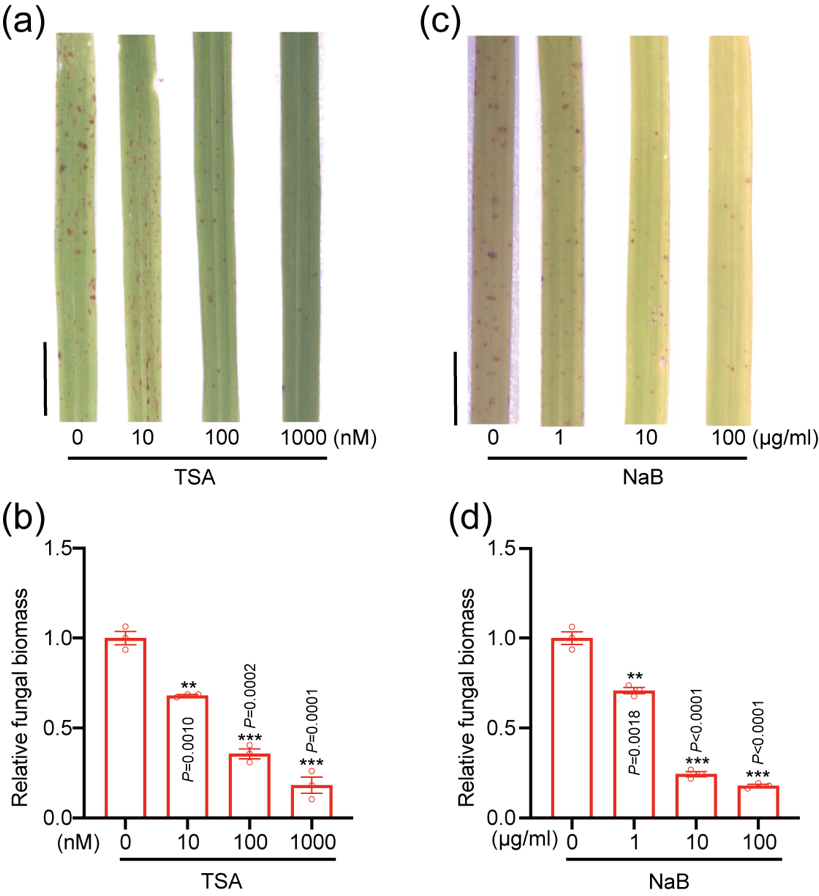
**

**Figure S7. Rice blast disease symptoms after treatment with HDAC inhibitors.**

(a, c) Disease symptoms after spray-inoculation with a spore suspension of *M. oryzae* for 5 dpi in WT treated with a series of concentrations of HDAC inhibitors including TSA (a) and NaB (c). Nipponbare without HDAC inhibitor treatment used as a control (Mock). Scale bar = 1 cm. (b, d) Relative fungal biomass after spray-inoculation with a spore suspension of *M. oryzae* for 5 dpi in WT treated with a series of concentrations of HDAC inhibitors including TSA (b) and NaB (d), which was determined using qPCR for the *MoPot2* gene normalized to rice *Ubiquitin*. Fungal biomass in the Nipponbare plants infected with *M. oryzae* without HDAC inhibitor treatment were set to 1.00. Values are means ± SEM (*n* = 3). Experiments were repeated three times with similar results. Asterisks mark significant changes compared with Mock (Nipponbare without HDAC inhibitor treatment) based on Student’s *t* test: ***P*<0.01, ****P*<0.001.


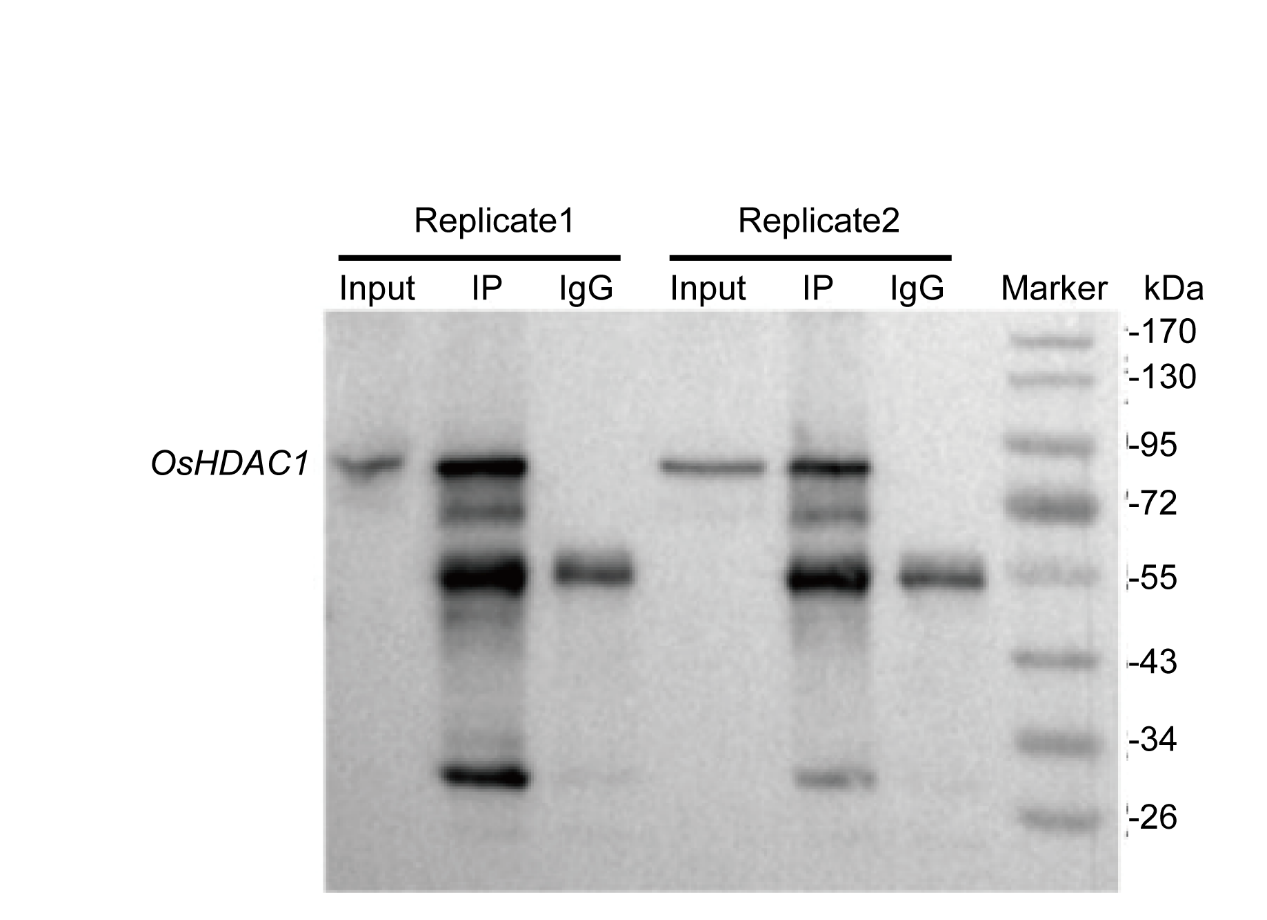


**Figure S8. Specificity testing of the anti-HA antibody for chromatin immunoprecipitation sequencing (ChIP-Seq) analysis in OsHDAC1 OE5 plants.** Molecular weights of proteins (kDa) are shown on the Right. IP, immunoprecipitation.


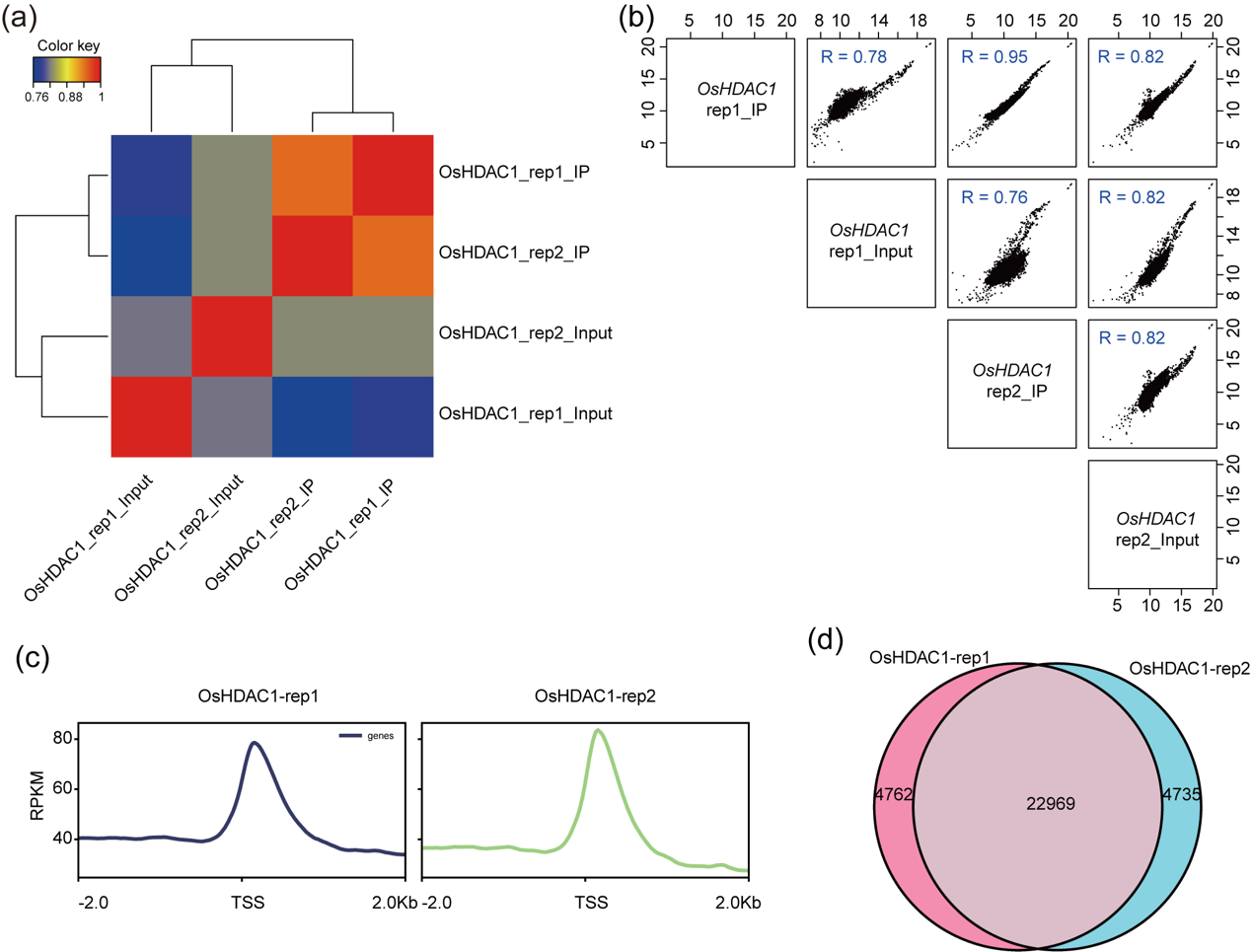


**Figure S9. The correlation analysis of the two biological replicates of OsHDAC1 in ChIP-seq.**

(a) Heatmap showing the high correlation between two biological replicates in ChIP-seq experiments. The color scale from blue to red represents the lower and higher inter sample correlations obtained through gene expression levels, respectively. (b) Scatter plot showing the high correlation between two biological replicates in ChIP-seq experiments. The horizontal and vertical coordinates represent the log10 (RPKM) of different sample genes, respectively. (c) Profile plot shows the high reproducibility of the mean density of OsHDAC1 occupancy at genome sites from the two biological replicates. (d) Profile plot shows the high reproducibility of the mean density of OsHDAC1 occupancy at all OsHDAC1-occupied sites from the two biological replicates.

**
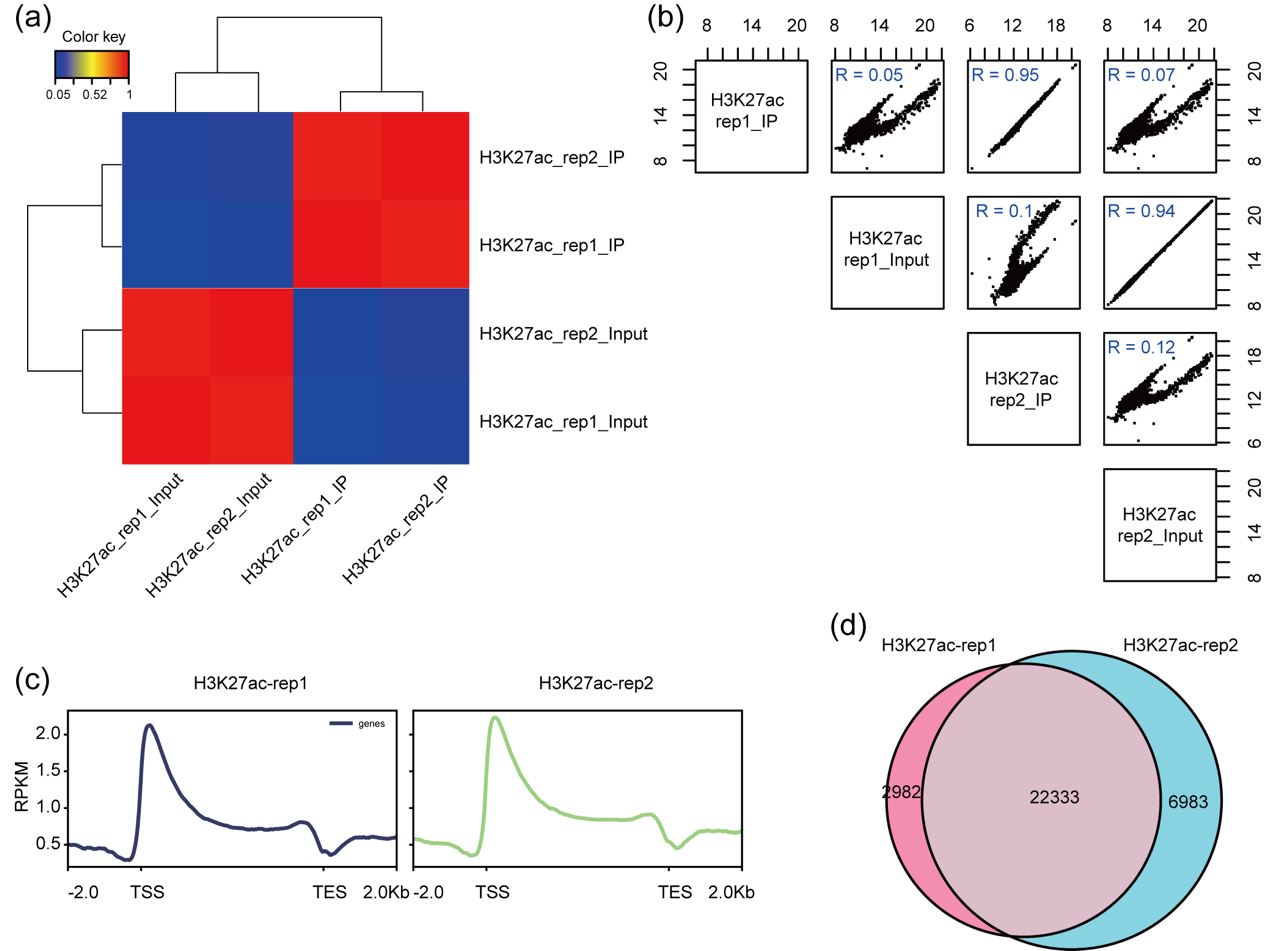
**

**Figure S10. The correlation analysis of the two biological replicates of H3K27ac in ChIP-seq.**

A. Heatmap showing the high correlation between two biological replicates in ChIP-seq experiments. The color scale from blue to red represents the lower and higher inter sample correlations obtained through gene expression levels, respectively. B. Scatter plot showing the high correlation between two biological replicates in ChIP-seq experiments. The horizontal and vertical coordinates represent the log10 (RPKM) of different sample genes, respectively. C. Profile plot shows the high reproducibility of the mean density of H3K27ac occupancy at genome sites from the two biological replicates. D. Profile plot shows the high reproducibility of the mean density of H3K27ac occupancy at all H3K27ac-occupied sites from the two biological replicates.


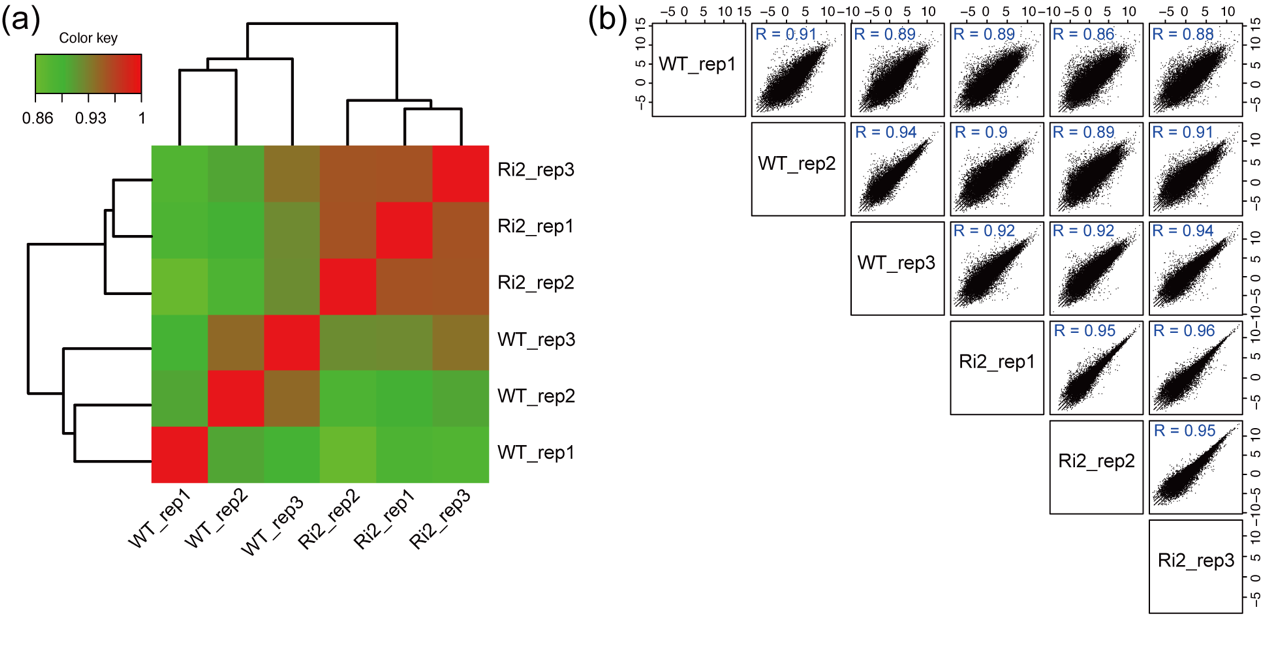


**Figure S11. The correlation analysis of the three biological replicates of OsHDAC1 in RNA-seq.**

(a) Heatmap showing the high correlation between three biological replicates in RNA-seq experiments. The color scale from blue to red represents the lower and higher inter sample correlations obtained through gene expression levels, respectively. (b) Scatter plot showing the high correlation between three biological replicates in RNA-seq experiments. The horizontal and vertical coordinates represent the log10 (RPKM) of different sample genes, respectively.


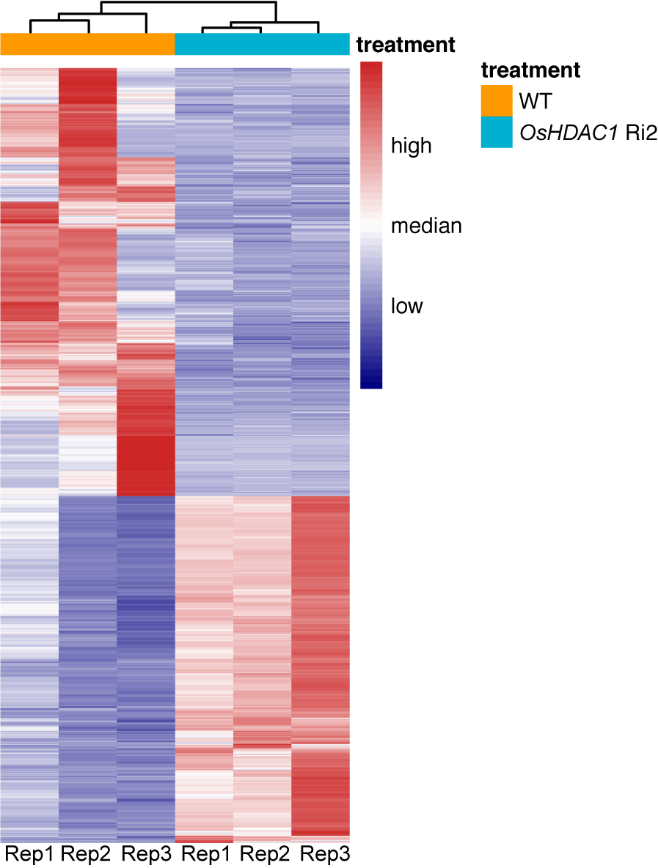


**Figure S12. Heatmap showing different expression of numerous DEGs in rice seedlings between the *OsHDAC1* Ri2 line and WT.**

Color bar on the top indicates the Z-score.


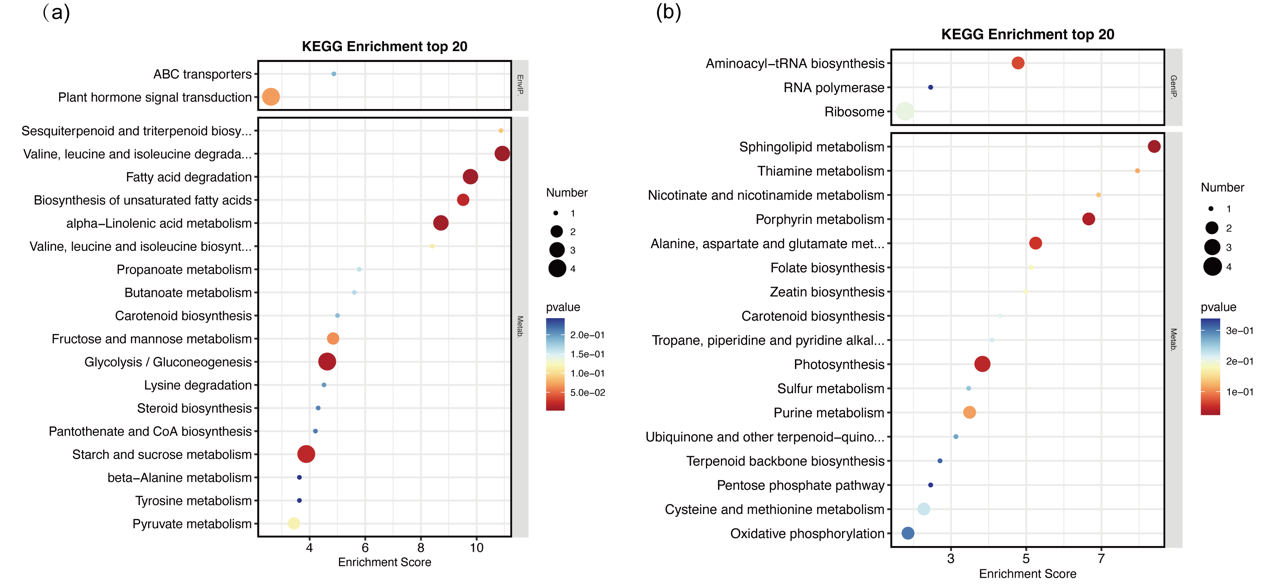


**Figure S13. Significantly enriched KEGG terms among OsHDAC1-regulated DEGs.**

(a) KEGG results showing the related biological pathway enriched from downregulated DEGs in *OsHDAC1* Ri2 line compared with WT. (b) KEGG results showing the related biological pathway enriched from upregulated DEGs in *OsHDAC1* Ri2 line compared with WT.

**Table S1. Primers used in this study.**

| **Primer Name** | **Sequence (5’-3’)** | **Vector name** |
| --- | --- | --- |
| **For yeast two hybrid assays** | | |
| OsHDAC1-F | atggccatggaggccgaattcATGGACGCCTCCGCCGGA | pGBK-T7 |
| OsHDAC1-R | tcgacggatccccgggaattcTTATGTCTTCTGGTGCACCGATG |  |
| OsHDAC1 N-ter-F | atggccatggaggccgaattcATGGACGCCTCCGCCGGA |  |
| OsHDAC1 N-ter-R | tcgacggatccccgggaattcCTTGGCCCCGTCGGCCCC |  |
| OsHDAC1 HD-F | atggccatggaggccgaattcCGGCGGGTGTGCTACTTCT |  |
| OsHDAC1 HD -R | tcgacggatccccgggaattcCTCAGGGGGTCGCTCTTGA |  |
| OsHDAC1 C-ter -F | atggccatggaggccgaattcGCTGAGCTACCTGAGCAAGATGA |  |
| OsHDAC1 C-ter -R | tcgacggatccccgggaattcTTATGTCTTCTGGTGCACCGATG |  |
| OsGRAS30-F | gccatggaggccagtgaattcATGGCTCAGTTCGGCGGC | pGAD-T7 |
| OsGRAS30-R | atgcccacccgggtggaattcTCACTGCATGATTTGGTTGAGAG |  |
| OsGRAS30 N-ter -F | gccatggaggccagtgaattcATGGCTCAGTTCGGCGGC |  |
| OsGRAS30 N-ter -R | atgcccacccgggtggaattcGGCGCACGTCACCATGAG |  |
| OsGRAS30 N-ter -F | gccatggaggccagtgaattcGGCGCCATCCAGGCGGGC |  |
| OsGRAS30 N-ter -R | atgcccacccgggtggaattcGTACAGCCTCTGGCTATGCCA |  |
| OsGRAS30 C-ter -F | gccatggaggccagtgaattcTCGGCGTCGGCGTGGCGC |  |
| OsGRAS30 C-ter -R | atgcccacccgggtggaattcTCACTGCATGATTTGGTTGAGAG |  |
| **For split luciferase complementation assay** | | |
| OsHDAC1-F | acgggggacgagctcggtaccATGGACGCCTCCGCCGGA | p1300-nLUC |
| OsHDAC1-R | cgcgtacgagatctggtcgacTGTCTTCTGGTGCACCGATGG |  |
| OsGRAS30-F | tacgcgtcccggggcggtaccATGGCTCAGTTCGGCGGC | p1300-cLUC |
| OsGRAS30-R | atttgttggatcccgggtaccTCACTGCATGATTTGGTTGAGAG |  |
| **For subcellular localization assay** | | |
| OsGRAS30-F | acgggggactctagaggatccATGGCTCAGTTCGGCGGC | pBI121 |
| OsGRAS30-R | cgatcggggaaattcgagctcTTACTTGTACAGCTCGTCCATGCC |  |
| **For protein purification** | | |
| OsHDAC1-F | ccgcgtggatccccggaattcATGGACGCCTCCGCCGGA | pGEX-4T1 |
| OsHDAC1-R | ctcgagtcgacccgggaattcTTATGTCTTCTGGTGCACCGATG |  |
| OsHDAC1-F | gctgatatcggatccgaattcATGGACGCCTCCGCCGGA | pET-32a |
| OsHDAC1-R | ttgtcgacggagctcgaattcTGTCTTCTGGTGCACCGATGG |  |
| OsGRAS30-F | gagggaaggatttcagaattcATGGCTCAGTTCGGCGGC | pMal-c2X |
| OsGRAS30-R | gactctagaggatccgaattcCTGCATGATTTGGTTGAGAGCA |  |
| **For construction of overexpression transgenic rice plants** | | |
| OsGRAS30-F | cagcccgggggatccccaatactATGGCTCAGTTCGGCGGC | pCXUN |
| OsGRAS30-R | aacccgctgttatccccaatactCTGCATGATTTGGTTGAGAGCA |  |
| **For construction of mutant transgenic rice plants** | | |
| OsGRAS30-gRT1+ | AGGGACTAGAGACGCGTACAgttttagagctagaaat | pYLCRISPR/Cas9Pubi-H  pYLsgRNA-OsU6 |
| OsGRAS30-U6aT1- | TGTACGCGTCTCTAGTCCCTggcagccaagccagca |  |
| OsGRAS30-gRT2+ | CCTGCGGTCGGTGAATCTCAgttttagagctagaaat |  |
| OsGRAS30-U6bT2- | TGAGATTCACCGACCGCAGGaacacaagcggcagc |  |
| U-F | CTCCGTTTTACCTGTGGAATCG |  |
| gR-R | CGGAGGAAAATTCCATCCAC |  |
| Pps-GGL | TTCAGAGGTCTCTCTCGACTAGTATGGAATCGGCAGCAAAGG |  |
| Pgs-GG2 | AGCGTGGGTCTCGTCAGGGTCCATCCACTCCAAGCTC |  |
| Pps-GG2 | TTCAGAGGTCTCTCTGACACTGGAATCGGCAGCAAAGG |  |
| Pgs-GGR | AGCGTGGGTCTCGACCGACGCGTATCCATCCACTCCAAGCTC |  |
| **For mutant site detecting:** | | |
| *osgras30*-F | CGAGAGCGACCACCCATTC | |
| *osgras30*-R | GCGCGGACTGCGGGAAC | |
| **For RT-qPCR** | | |
| Ubiquitin-F | AACCAGCTGAGGCCCAAGA | |
| Ubiquitin-R | ACGATTGATTTAACCAGTCCATGA | |
| OsHDAC1-F | AGATGCCGCAGATCAGGATG | |
| OsHDAC1-R | AGGGAGCCTGGTTCGTCTAT | |
| OsWRKY45-F | CGGGTAAAACGATCGAAAGA | |
| OsWRKY45-R | TTTCGAAAGCGGAAGAACAG | |
| MoPot2-F | ACGACCCGTCTTTACTTATTTGG | |
| MoPot2-R | AAGTAGCGTTGGTTTTGTTGGAT | |
| OsRLR1-F | TTCCGGAAGAATGACCGCAA | |
| OsRLR1-R | CGGATTGTCTCAACCAGGCT | |
| OsRGA5-F | TGGATGCTCCGGTGAGTTTC | |
| OsRGA5-R | TATCTTGCCCTGTGGCTTGG | |
| OsSSI2-F | ACTCTCGATGGTGTCCGAGA | |
| OsSSI2-R | TAGGGTCCATTCCAGAGCCA | |
| OsF3H-F | CACCGCTACCTCCCTGATTG | |
| OsF3H-R | CAGGCTCTCGGATATCGCTC | |
| OsGRAS30-F | GGATCACAACAAGTCGTCGCT | |
| OsGRAS30-R | AGTCGAACATGGAGGCGTAGT | |
| **For ChIP-qPCR** | | |
| Ubiquitin-F | TCGGAGACCGTGCTAGGTTT | Anti-HA and H3K27ac antibody |
| Ubiquitin-R | GCCAGCGCCCATCGATT |  |
| OsRLR1-F | AAAGAAACCGTCAAACGCCG |  |
| OsRLR1-R | TCCGCACCTACAAAATGATTAAATA |  |
| OsRGA5-F | ACCATGTTTTCGATGCGCTG |  |
| OsRGA5-R | TCTGCGTCTTGGAGAAACCG |  |
| OsSSI2-F | GGATTGGTTCCCCGTGTGT |  |
| OsSSI2-R | AGACGAGGCAAGCAAAACGA |  |
| OsF3H-F | GCCTGACTACGCTTCTCCG |  |
| OsF3H-R | CGGTACACACACACAACTTGG |  |
